# Bellidifolin Ameliorates Isoprenaline-Induced Myocardial Fibrosis by Regulating TGF-β1/Smads and p38 Signaling and Preventing NR4A1 Cytoplasmic Localization

**DOI:** 10.3389/fphar.2021.644886

**Published:** 2021-04-30

**Authors:** Hong-Xia Yang, Jia-Huan Sun, Ting-Ting Yao, Yuan Li, Geng-Rui Xu, Chuang Zhang, Xing-Chao Liu, Wei-Wei Zhou, Qiu-Hang Song, Yue Zhang, Ai-Ying Li

**Affiliations:** ^1^ Department of Biochemistry and Molecular Biology, College of Basic Medicine, Hebei University of Chinese Medicine, Shijiazhuang, China; ^2^ Department of Clinical Foundation of Chinese Medicine, College of Basic Medicine, Hebei University of Chinese Medicine, Shijiazhuang, China; ^3^ Department of Medical Laboratory Science, College of Integration of Chinese and Western Medicine, Hebei University of Chinese Medicine, Shijiazhuang, China; ^4^ Hebei Higher Education Institute Applied Technology Research Center on TCM Formula Preparation, Shijiazhuang, China; ^5^ Hebei Key Laboratory of Chinese Medicine Research on Cardio-cerebrovascular Disease, Shijiazhuang, China

**Keywords:** bellidifolin, Gentianella acuta, myocardial fibrosis, TGF-β1/smads pathway, p38, orphan nuclear receptor NR4A1

## Abstract

Myocardial fibrosis is closely related to high morbidity and mortality. In Inner Mongolia, *Gentianella amarella subsp. acuta* (Michx.) J.M.Gillett (*G. acuta*) is a kind of tea used to prevent cardiovascular diseases. Bellidifolin (BEL) is an active xanthone molecule from *G. acuta* that protects against myocardial damage. However, the effects and mechanisms of BEL on myocardial fibrosis have not been reported. *In vivo*, BEL dampened isoprenaline (ISO)-induced cardiac structure disturbance and collagen deposition. *In vitro,* BEL inhibited transforming growth factor (TGF)-β1-induced cardiac fibroblast (CF) proliferation. *In vivo and in vitro*, BEL decreased the expression of α-smooth muscle actin (α-SMA), collagen Ⅰ and Ⅲ, and inhibited TGF-β1/Smads signaling. Additionally, BEL impeded p38 activation and NR4A1 (an endogenous inhibitor for pro-fibrogenic activities of TGF-β1) phosphorylation and inactivation *in vitro*. In CFs, inhibition of p38 by SB203580 inhibited the phosphorylation of NR4A1 and did not limit Smad3 phosphorylation, and blocking TGF-β signaling by LY2157299 and SB203580 could decrease the expression of α-SMA, collagen I and III. Overall, both cell and animal studies provide a potential role for BEL against myocardial fibrosis by inhibiting the proliferation and phenotypic transformation of CFs. These inhibitory effects might be related to regulating TGF-β1/Smads pathway and p38 signaling and preventing NR4A1 cytoplasmic localization.

## Introduction

Myocardial fibrosis is a morphological feature of cardiac remodeling resulting from myocardial infarction (MI) (Masci et al., 2013; Di Bello et al., 2017). It is closely related to high morbidity and mortality (Leyva et al., 2012). It is well known that cardiac fibroblasts (CFs) play a critical role in the development of the fibrotic response and pathological cardiac remodeling (Ivey et al., 2018). Following myocardial injury, CFs are involved in the post MI inflammatory and fibrotic response (Ivey et al., 2018). CFs undergo proliferation and phenotypic transformation to myofibroblasts with high expression of α-smooth muscle actin (α-SMA) (Song et al., 2020), which secretes and deposits many extracellular matrix (ECM) proteins including collagens (in particular, type Ⅰ and type Ⅲ) in cardiac tissue (van Nieuwenhoven and Turner, 2013). Excessive accumulation of ECM is characteristic of myocardial fibrosis, which increases ventricle wall stiffness to disturb cardiac function and architecture leading to heart failure (Nielsen et al., 2019). Thus, the reduction of the proliferation and phenotypic transformation of CFs may be a potential treatment strategy for myocardial fibrosis.

Transforming growth factor (TGF)-β1 is a multifunctional cytokine with a broad range of effects on cell proliferation, differentiation, migration, and apoptosis (Stewart et al., 2018; Xu et al., 2018; Zhang et al., 2018). TGF-β takes part in myocardial fibrosis by activating TGF-β-receptors (TβRs). The TβRs then mediate the downstream molecules such as Smads2, 3, and 4 (Khalil et al., 2017). Smad3 plays the most essential role in TGF-β1-mediated signal transduction, and is a critical mediator of myofibroblast differentiation (Datto et al., 1999; Khalil et al., 2017). Therefore, inhibition of the TGF-β/Smads pathway, especially TGF-β/Smad3, is a promising therapeutic approach for myocardial fibrosis.

The p38 mitogen-activating protein kinases (MAPK) can also be activated by TGF-β1. Research has shown that p38 in CFs played a core regulatory role in myocardial fibrosis (Muslin, 2008; Molkentin et al., 2017). A report showed that activin A promoted CFs proliferation and collagen production by activating p38 signaling while p38 inhibitor (SB203580) inhibited activin A-regulated responses (Hu et al., 2016). Inhibition of p38 prevented TGF-β-induced fibroblasts activation and alleviated collagen synthesis (Dolivo et al., 2019). Thus, inhibition of p38 signaling may be an effective therapeutic strategy for preventing CFs phenotypic transformation.

However, how p38 signaling participates in the regulation of myocardial fibrosis is not fully understood. Researchers have demonstrated that the activation of p38 could increase the Smad3 phosphorylation in hepatic fibrosis (Fabre et al., 2018). However, the role of p38 signaling in phenotypic transformation of CFs remains elusive. Orphan nuclear receptor NR4A1 is an endogenous inhibitor of transforming growth factor-β (TGF-β) signaling, which was phosphorylated and inactivated and transferred from the nucleus to the cytoplasm in fibrotic diseases (Palumbo-Zerr et al., 2015). Prevention of p38 signaling impeded NR4A1 cytoplasmic localization in the study of α-lipoic acid (ALA)-induced apoptosis of vascular smooth muscle cells (VSMCs) (Kim et al., 2010). Thus, this study explored how p38 is involved in regulating phenotypic conversion of CFs to myofibroblasts.

*Gentianella amarella subsp. acuta* (Michx.) J.M.Gillett (*G. acuta*), belonging to the Gentianaceae, was called “guixincao” in the Hulunbeier districts of Inner Mongolian. In these regions, Ewenki people keep a habit of drinking “guixincao” tea, and employ it to treat angina by water decoction or chewing for a long time (Wunir et al., 2009; Li et al., 2010). Our previous studies demonstrated that TGF-β1 was elevated in isoprenaline (ISO)-induced cardiac fibrotic tissue, and *G. acuta* ameliorated myocardial fibrosis by inhibiting TGF-β1/Smads signaling (Li et al., 2019; Yang et al., 2020).

**FIGURE 1:**
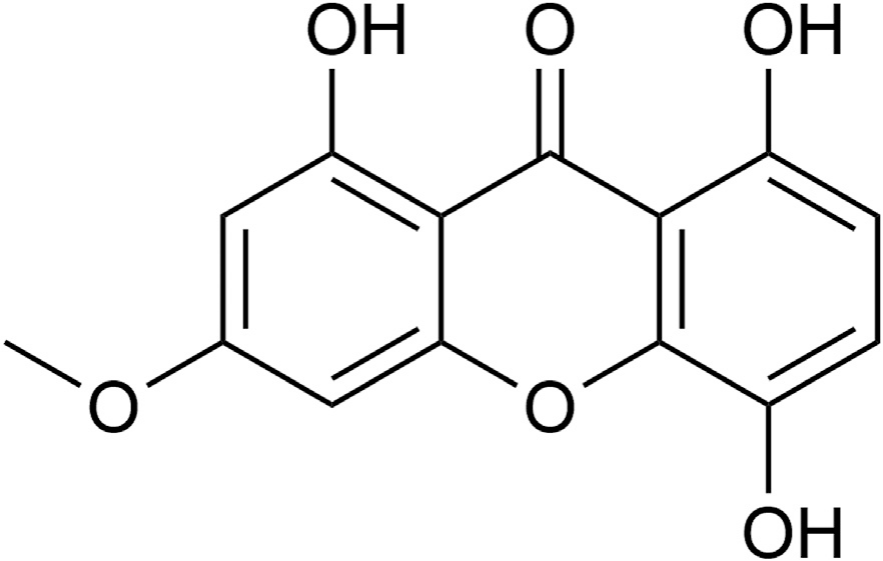
Chemical structure of Bellidifolin (BEL).

Bellidifolin (Figure 1) is a xanthone reported to protect the heart, which is abundant in *G. acuta* (Wang et al., 2017; Ren et al., 2019). BEL decreases myocardial ischemia-reperfusion injury *in vivo* and protects cardiomyocytes from oxidative damage *in vitro* (Wang et al., 2017; Wang et al., 2018; Ren et al., 2019). However, the effect and associated mechanism of BEL on the proliferation and phenotypic transformation of CFs in the progression of myocardial fibrosis remained unclear. Here, we show that BEL can inhibit myocardial fibrosis by suppressing the proliferation and phenotypic transformation of CFs. The inhibitory mechanism of BEL on myocardial fibrosis was verified by regulating the TGF-β/Smads and p38 pathway and preventing NR4A1 cytoplasmic localization. Therefore, BEL would be a potential therapeutic agent for ameliorating myocardial fibrosis.

## Materials and Methods

### Primary Drugs, Antibodies and Reagents

BEL was acquired from Chengdu Alfa Biotechnology (China, with 98% purity by HPLC). Trimetazidine (TMZ) was procured from Nanjing Zenkom Pharmaceutical Co., Ltd. (China). ISO was obtained from Tokyo Chemical Industry (Japan). Antibodies for TβRⅠ (cat. no. GB11271), Histone H3 (cat. no. GB13102–1) and α-SMA for immunofluorescence analysis (cat. no. GB13044) were derived from Wuhan Servicebio (China). Antibodies for α-SMA used for western blotting detection (cat. no. ab32575), Smad2 (cat. no. ab40855), phospho-Smad2 (S467) (cat. no. ab53100), Smad3 (cat. no. ab40854), phospho-Smad3 (S423 + S425) (cat. no. ab52903), and Smad4 (cat. no. ab40759) were purchased from Abcam Technology (United Kingdom). Antibodies for p38 and phospho-p38 (Thr180/Tyr182) (cat. no. 8203) were purchased from CST (United States). The antibody for TβRⅡ (cat. no. A11788) was purchased from ABclonal Technology (United States). Antibodies for collagen Ⅰ (cat. no. AF7001), collagen Ⅲ (cat. no. AF0136), phospho-TβRⅠ (Thr204) (cat. no. AF8080), and phospho-TβRⅡ (Tyr284) (cat. no. AF8191) were purchased from Affinity Biosciences (United States). The antibody for NR4A1 (cat. no. abs137327) was obtained from Absin (China). The antibody for phospho-NR4A1 (Ser351) (cat. no. orb191558) was derived from Biorbyt Ltd. (United Kingdom). The antibody for Glyceraldehyde 3-phosphate dehydrogenase (GAPDH; cat. no. 60004-1-Ig) was gained from Wuhan Proteintech (China). HRP-conjugated anti-rabbit and anti-mouse IgG (cat. nos. ZdR-5306 and ZdR-5307) antibodies were obtained from Beijing ZSGB Bioengineering Institute (China). Goat-anti-mouse-TRITC and goat-anti-rabbit-Alexa-488 were gained from Wuhan Proteintech (China). The 4′, 6-diamidino-2-phenylindole (DAPI) was purchased from Wuhan Servicebio (China). Recombinant human TGF-β1 was acquired from Gibco (United States). SB203580 (p38 inhibitor) and LY2157299 (TβRⅠ inhibitor) were obtained from MedChem Express (United States).

### Animal Model of Myocardial Fibrosis

Male mice (18–22 g) were purchased from the Experimental Animal Center of Hebei Medical University (Shijiazhuang, China) and kept in a 12 h light/12 h dark cycle with free access to food and water. All experiments were according to the People’s Republic of China legislation for the use and care of laboratory animals. The animal care and study protocol were authorized by the Institutional Animal Care and Use Committee of Hebei University of Chinese Medicine (no. DWLL201802).

Mice were subjected to ISO injection to induce myocardial fibrosis after MI as described previously (Yang et al., 2017; Koga et al., 2019). Briefly, ISO (5 mg/kg/day) was injected subcutaneously into the mice for 7 days. The mice were then randomly assigned into five groups: control group (Control), model group (ISO), BEL intervention group (ISO + BEL 25 and 50 mg/kg/day, p. o., BEL 25 and 50 mg/kg), TMZ intervention group (ISO + TMZ 20 mg/kg/day, p. o., TMZ 20 mg/kg). The drug interventions were administrated for 21 days from the second day of the experiment. On day 22, the mice were anesthetized with 0.3% sodium phenobarbital and then sacrificed. The heart tissues were fixed with 4% paraformaldehyde for histopathological analyses, and the residual heart tissues were then frozen at −80°C for western blotting detection.

### Morphological Detection of Heart

The heart tissues were embedded in paraffin wax after fixing in 4% paraformaldehyde for 24 h. Paraffin-embedded heart tissues were cut into 5 µm sections. Hematoxylin and eosin (HE; Sigma, United States) and Masson trichrome (Servicebio, China) were used to observe cardiac histopathological changes according to the manufacturer’s protocol. Briefly, cardiac sections were dewaxed to water and stained. Sections were then dehydrated, made transparent, and sealed. The morphological changes were observed and photographed using light microscopy (Leica, Germany).

### Primary CFs Isolation and Culture

As per the previously mentioned method, CFs were isolated from 1 to 3 days old neonatal SD rats under sterile conditions by differential adherent method (Olson et al., 2005). Briefly, the hearts were aseptically removed from the neonatal rats and minced into small pieces with eye scissors in ice-cold PBS buffer (pH 7.4). The minced ventricles were digested for 5 min with 0.7 mg/ml collagenase Ⅱ (Solarbio, China) at 37°C and then the digestive liquid was then discarded. Next, the tissue fragments were further dissociated for several 5 min with 0.08% trypsin (Gibco, United States) without EDTA at 37°C. The digestion was repeated for 5–7 times until the fragments were completely digested. The cell suspension was then centrifugated, resuspended, and inoculated in culture bottles. After 1 h, the non-adherent cells were discarded, and the adherent cells were cultured in DMEM medium (Gibco, United States) supplemented with 10% fetal bovine serum (BI, Israel), 100 unit/ml penicillin (BI, Israel), and 100 μg/ml streptomycin (BI, Israel) in a humidified atmosphere containing 5% CO_2_ at 37°C. CFs were identified by Vimentin immunofluorescence.

### CF Grouping and Drug Administration

BEL was dissolved in DMSO, and the final concentration of DMSO (Solarbio, China) is 1/1,000. The CFs were divided into six groups: control, TGF-β1, TGF-β1 with 1‰ DMSO, TGF-β1 plus BEL 15, 30, and 60 µM groups. The CFs were pretreated with DMSO or BEL (15, 30, and 60 µM) for 0.5 h and then stimulated with TGF-β1 (10 ng/ml) for an additional 24 h.

SB203580 and LY2157299 were dissolved in DMSO, and CFs were incubated with DMSO, BEL (60 µM), SB203580 (0.5 µM), LY2157299 (10 µM), or SB203580 plus LY2157299 for 0.5 h. The samples were then administrated with TGF-β1 (10 ng/ml) for an additional 24 h.

### CCK-8 Assays

CFs were inoculated into 96-well plates and grown to 70% confluence. Cells were cultured in a serum free medium for 18 h followed by administration with BEL (7.5, 15, 30, 60, and 120 μM) in the absence or the presence of TGF-β1 for 24 h. The cell viability was detected by CCK-8 kit (MedChem Express, United States). CCK-8 solution (20 μL) was added into the wells, and CFs were incubated for 3 h at 37°C. The OD values of each well were detected by Varioskan LUX Multimode Microplate Reader at the absorbance of 450 nm (Thermo Fisher Scientific, United States).

### Cell Immunofluorescence Staining

Immunofluorescence staining was performed to detect the expression of α-SMA, collagen Ⅰ, collagen Ⅲ, Smads2, 3, 4, NR4A1, phospho-Smads2, 3, and phospho-NR4A1. Briefly, cell climbing pieces were fixed in 4% paraformaldehyde and were permeabilized with 0.1% Triton X-100 for 20 min at room temperature (RT). The cells were subsequently blocked with 3% BSA for 90 min and then incubated with primary antibodies against α-SMA (1:500), collagen Ⅰ (1:100), collagen Ⅲ (1:100), Smad2 (1:50), Smad3 (1:50), Smad4 (1:50), NR4A1 (1:100), phospho-Smad2 (1:50), phospho-Smad3 (1:50), and phospho-NR4A1 (1:100) overnight in a humidity chamber at 4°C. Cells were subsequently incubated with species-specific secondary antibodies labeled with fluorescence for 1 h at 37°C. Cell nuclei were stained using DAPI. The sections were sealed with an anti-interferent fluorescent reagent. The cell slides were detected with Leica DM5000 B fluorescence microscope (Germany) or Leica SP8 confocal microscopy (Germany) using 40✕ or 63✕ magnification. Red fluorescence signal was measured at excitation and emission wavelengths of 550 and 570 nm. Green fluorescence signal was captured at excitation and emission wavelengths of 488 and 514 nm. DAPI was visualized at excitation and emission wavelengths of 360 and 461 nm. The results were quantified by Image Pro Plus 6.0 software (Media Cybernetics, United States).

### Western Blotting Assay

A western blotting assay was used to measure the expression of α-SMA, collagen Ⅰ, collagen Ⅲ, TβRⅡ, phospho-TβRⅡ, TβRⅠ, phospho-TβRⅠ, Smads2, 3, 4, phospho-Smads2, 3, p38, phospho-p38, NR4A1, and phospho-NR4A1; GAPDH was used as an internal reference, and Histone H3 was used as a nuclear internal reference. Tissue samples or cells were lyzed in RIPA buffer adding phenylmethyl sulfonyl fluoride (PMSF), protease inhibitor cocktail (Roche, Switzerland), and phosphatase inhibitors (Wuhan Servicebio, China). The nuclear and cytoplasmic proteins were extracted by Nuclear and Cytoplasmic Protein Extraction Kit (Shanghai Beyotime, China). The protein concentration was measured using a bicinchoninic acid (BCA) protein assay kit (Wuhan Servicebio, China). An equal amount of protein was separated using 10% SDS-PAGE and subsequently transferred from gel to polyvinylidene fluoride membranes. The membranes were interdicted with 5% defatted milk for 90 min at RT and individually incubated with primary antibodies α-SMA (1:5,000), collagen Ⅰ (1:1,000), collagen Ⅲ (1:1,000), TβRⅡ (1:1,000), phospho-TβRⅡ (1:3,000), TβRⅠ (1:1,000), phospho-TβRⅠ (1:3,000), Smad2 (1:2,000), Smad3 (1:10,000), Smad4 (1:5,000), phospho-Smad2 (1:1,000), phospho-Smad3 (1:2,000), p38 (1:1,000), phospho-p38 (1:1,000), NR4A1 (1:3,000), phospho-NR4A1 (1:500), GAPDH (1:10,000), and Histone H3 (1:10,000) at 4°C overnight. The membranes were incubated following the corresponding HRP-conjugated secondary antibody at RT for 60 min. The immunoreactive bands were examined with ECL (Invitrogen) reagents and the Fusion FX5 Spectra multifunction laser-scanning system (Vilber Lourmat, France). The protein expression was evaluated by ImageJ 3.0 software (National Institute of Health).

### Statistical Analysis

All data are expressed as the mean ± SEM. Statistical analyses were implemented with SPSS 21.0 statistical software. The significance of differences between groups were assessed using one-way ANOVA followed by Turkey's post hoc test and considered at a value of *p* < 0.05.

## Results

### BEL Ameliorates ISO-Induced Mouse Myocardial Fibrosis

**FIGURE 2:**
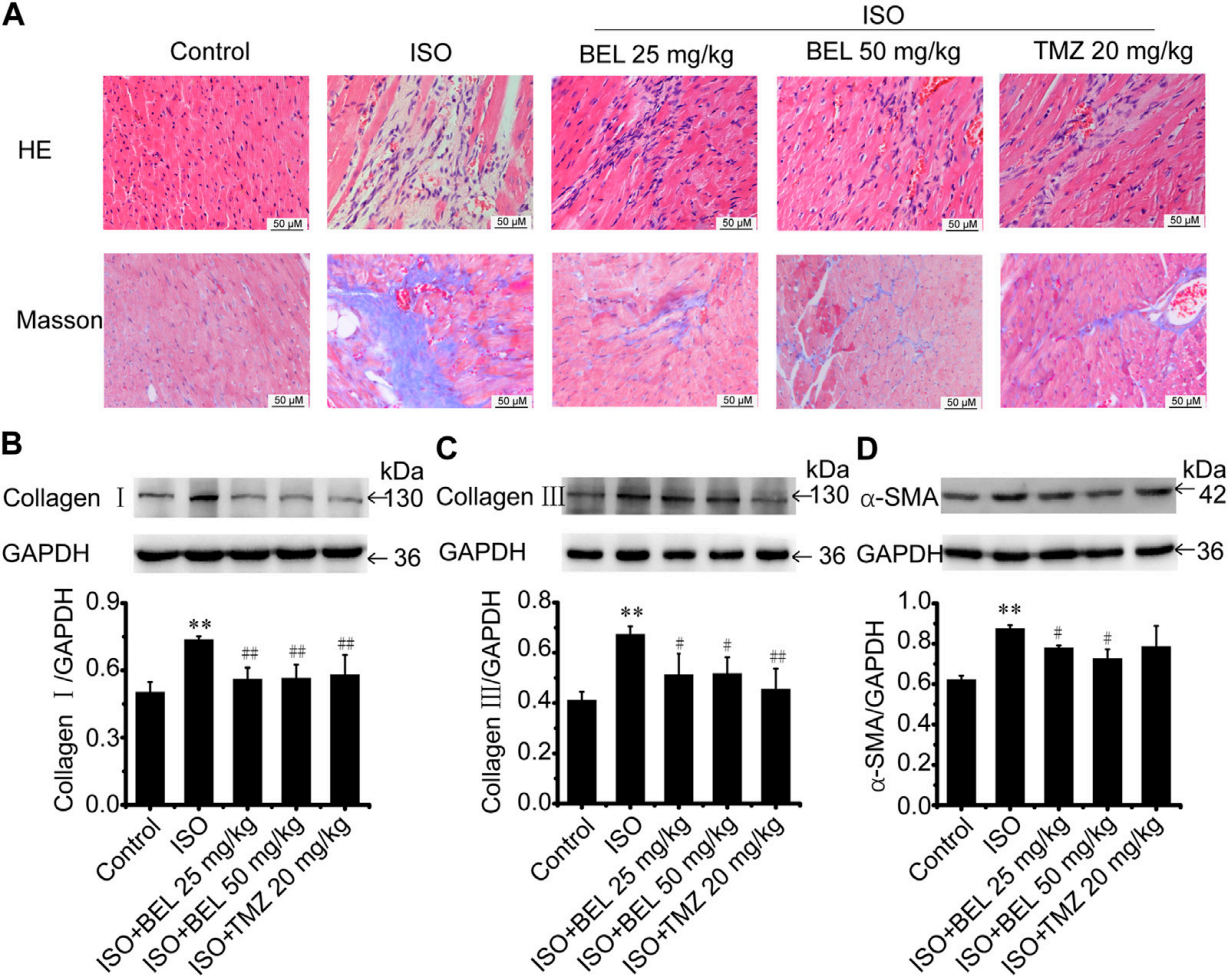
BEL ameliorates ISO-induced mouse myocardial fibrosis **(A)** HE staining to detect morphological changes; Masson trichrome staining to detect cardiac collagen deposition **(B–D)** Western blotting detection of collagen Ⅰ, collagen Ⅲ, and α-smooth muscle actin (α-SMA). Bar graphs show fold changes for the collagen Ⅰ, collagen Ⅲ, and α-SMA expression as analyzed by western blotting. Glyceraldehyde 3-phosphate dehydrogenase (GAPDH) was used as a loading control (*n* = 3). Data were shown as mean ± SEM. **p < 0.01 vs. control, #*p* < 0.05 vs. ISO, ##*p* < 0.01 vs. ISO.

A mouse model of myocardial fibrosis was established by subcutaneous injection of ISO (5 mg/kg/day) for 7 days. HE staining demonstrated that cardiac fibers and tissue structure were obvious in the control group; disturbed cardiac fibers and necrotic cardiomyocytes were present in the ISO group. Compared with the ISO group, treatments with BEL and TMZ dampened ISO-induced cardiac damages (Figure 2A). Masson trichrome staining determined that collagen deposition in myocardial tissues was significantly increased in the model group induced by ISO. However, collagen deposition was reduced in mouse hearts treated by BEL and TMZ compared with the ISO treated group (Figure 2A).

Western blotting was used to quantify the contents of collagen Ⅰ, and collagen Ⅲ—the primary compounds of ECM in fibrotic heart. The results revealed that the expression of cardiac collagen Ⅰ, and collagen Ⅲ were obviously elevated in the ISO group compared with the control group while ISO-induced elevations in collagen I, and collagen III expression were decreased by BEL and TMZ administration (Figures 2B,C).

Following myocardial injury, CFs transform to myofibroblast phenotype, which express contractile protein α-SMA, produce large amounts of collagen (in particular, collagen types I and III), and contribute to myocardial fibrosis (van Nieuwenhoven and Turner, 2013; Travers et al., 2016; Weber and Diez, 2016; Song et al., 2020). The level of α-SMA expression was examined by western blotting. The results demonstrated that α-SMA expression was significantly upregulated in mice with ISO-induced myocardial fibrosis compared with the control group. However, the upregulation of α-SMA expression induced by ISO was inhibited by BEL but not TMZ (Figure 2D). These results illustrated that BEL inhibited CFs activation, reduced collagen deposition, and ameliorated myocardial fibrosis.

BEL inhibits the proliferation and phenotypic transformation of CFs induced by TGF-β1.

**FIGURE 3:**
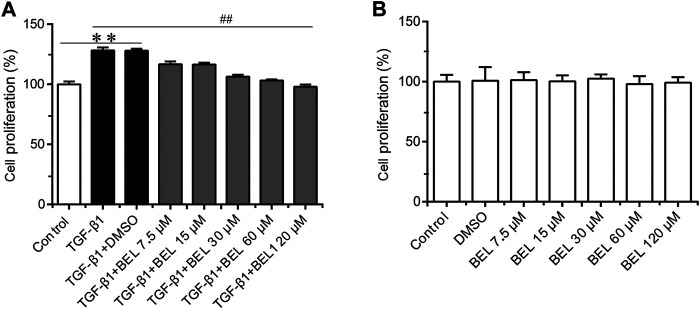
The effects of BEL on CFs proliferation in the presence or absence of TGF-β1 **(A)** The effects of different doses of BEL (7.5, 15, 30, 60, and 120 μM) on the proliferation of CFs induced by TGF-β1 using CCK-8 kit **(B)** The effects of different doses of BEL (7.5, 15, 30, 60, and 120 μM) on the viability of CFs without TGF-β1 stimulation using CCK-8 kit. Data were shown as mean ± SEM. **p < 0.01 vs. control, ^##^ *p* < 0.01 vs. TGF-β1.

It is well known that TGF-β1 contributes to myocardial fibrosis, and can induce the proliferation and activation of CFs. First, the CCK-8 kit was employed to detect the effect of BEL on TGF-β1-induced proliferation. CFs were pretreated with BEL (7.5, 15, 30, 60 and, 120 μM) for 0.5 h and then exposed to TGF-β1 (10 ng/ml) for 24 h. The results showed that the administration with BEL markedly inhibited CFs proliferation induced by TGF-β1 (Figure 3A). The role of BEL on CF viability without TGF-β1 stimulation was also investigated. CFs were administrated with BEL (7.5, 15, 30, 60, and 120 μM) for 24 h and examined by CCK-8 kit. The data showed that there were no significant differences by administrating BEL from 7.5–120 μM in comparison with the control group (Figure 3B). These results suggest that intervention with BEL at a concentration of 7.5–120 μM has no effect on CF viability while BEL administration could inhibit TGF-β1-induced CF proliferation.

**FIGURE 4:**
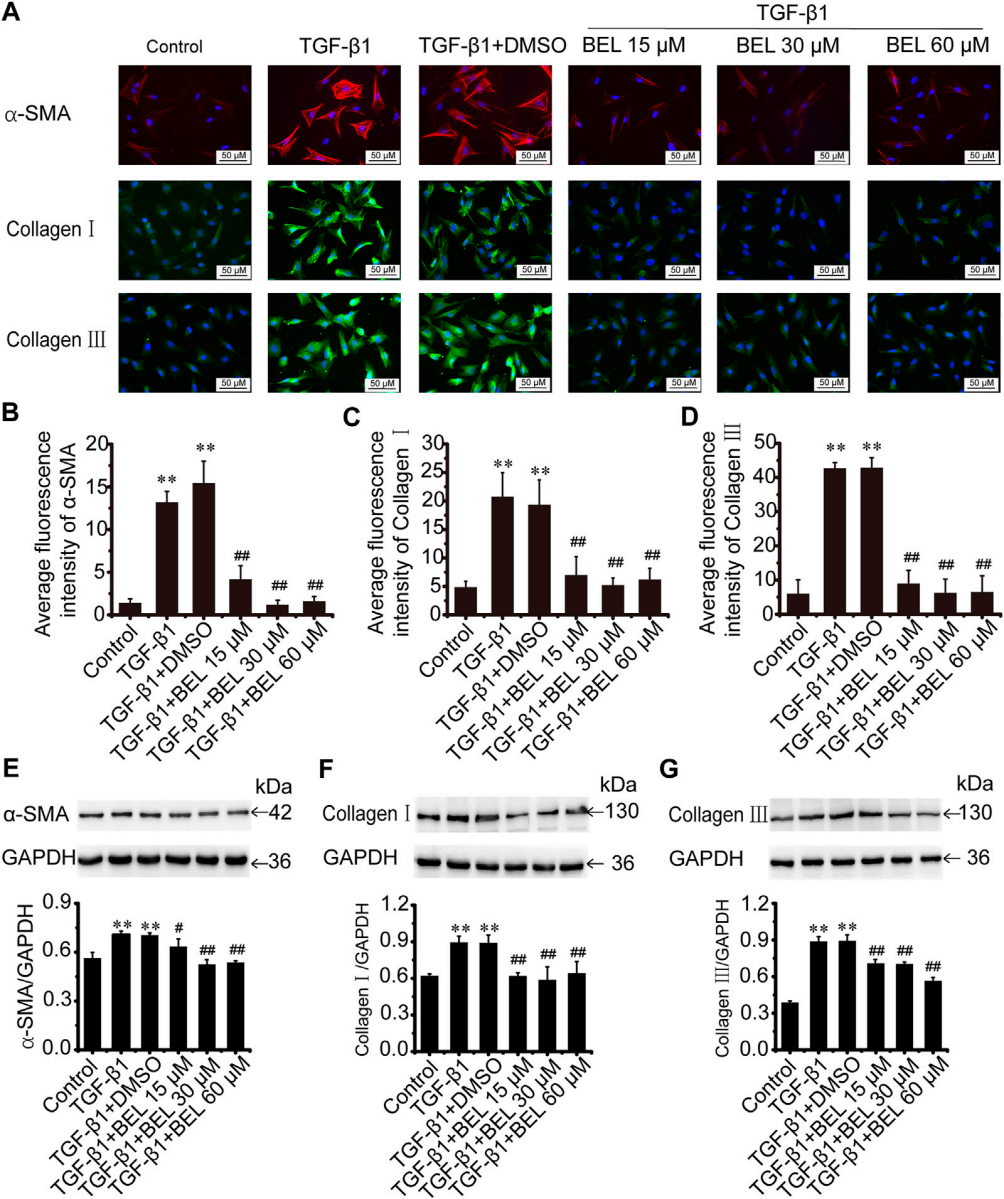
BEL decreases the expression of α-SMA, collagen Ⅰ, and collagen Ⅲ induced by TGF-β1 in CFs **(A)** The effects of different doses of BEL (15, 30, 60 μM) on α-SMA, collagen Ⅰ, and collagen Ⅲ expression induced by TGF-β1 using immunofluorescence staining. Cell nuclei was counterstained in blue with DAPI **(B–D)** Bar graphs show quantitative analyses of fluorescent signals for α-SMA, collagen I and collagen III **(E–G)** Western blotting detects the expression of α-SMA, collagen Ⅰ, and collagen Ⅲ expression. Bar graphs show fold changes of α-SMA, collagen Ⅰ, and collagen Ⅲ expression as analyzed by western blotting. GAPDH was used as a loading control (*n* = 3). Data were shown as mean ± SEM. **p < 0.01 vs. control, ^#^ *p* < 0.05 vs. TGF-β1, ^##^ *p* < 0.01 vs. TGF-β1.

The role of BEL on activated CFs induced by TGF-β1 was also investigated. The cultured CFs were exposed to TGF-β1 (10 ng/ml) in the absence or presence of BEL for 24 h. Immunofluorescence staining results revealed that the expression of α-SMA, collagen Ⅰ, and collagen Ⅲ were increased in CF stimulated with TGF-β1; these increases were decreased by BEL treatment (Figures 4A–D). Consistent with immunofluorescence staining, western blotting analysis further demonstrated that BEL decreased the expression of α-SMA, collagen Ⅰ, and collagen Ⅲ induced by TGF-β1 (Figures 4E–G). These results illustrated that BEL prevented TGF-β1-induced proliferation and activation of CFs.

### BEL Inhibits the Phosphorylation and Activation of TβR

**FIGURE 5:**
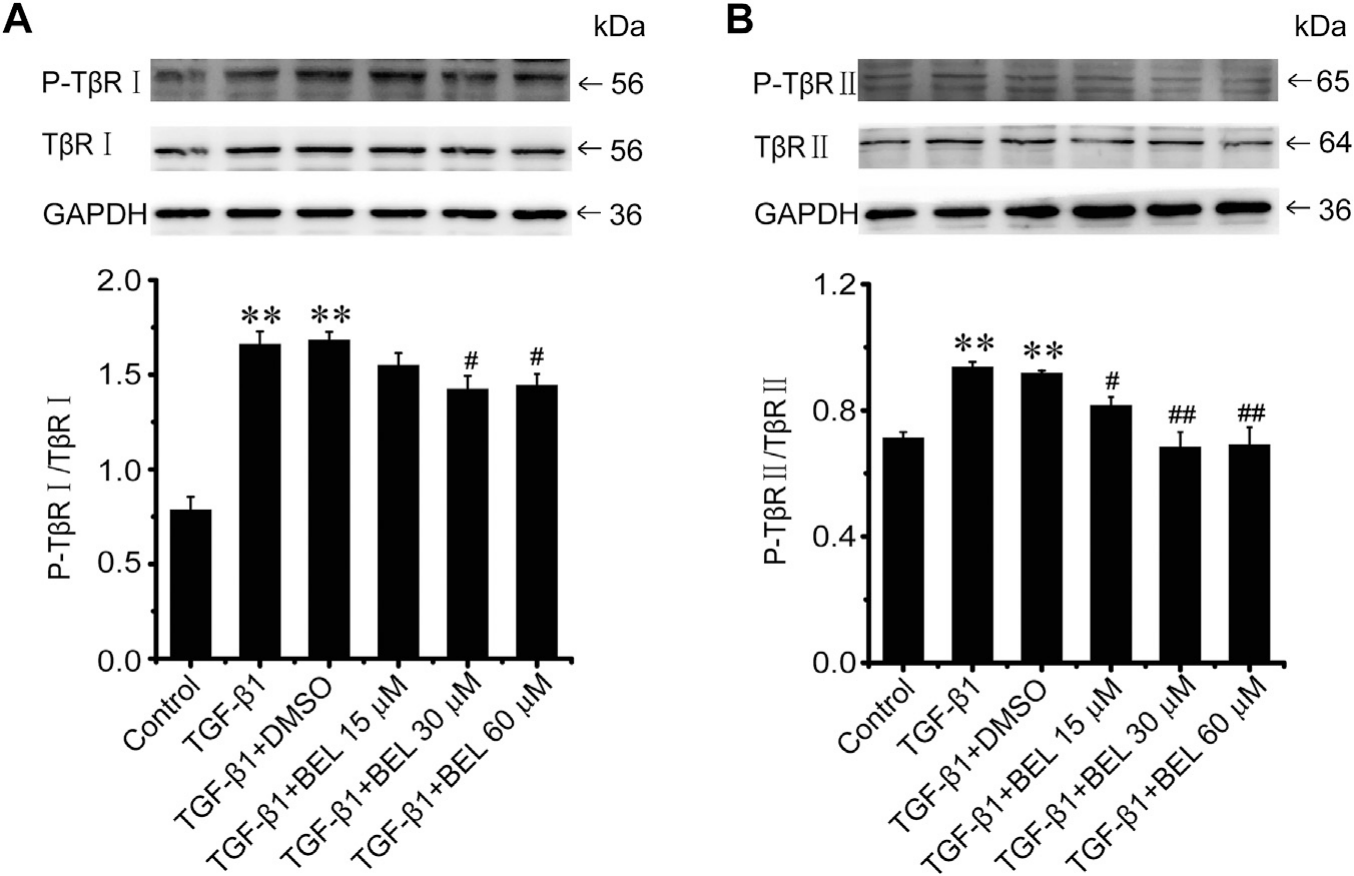
BEL suppresses the TβR phosphorylation induced by TGF-β1 in CFs **(A and B)** Western blotting detects TβRⅠ and TβRⅡ expression and phosphorylation level. Bar graphs show fold changes for the ratio of phosphorylated (P)-TβRI/TβRI, and P-TβRⅡ/TβRⅡ as analyzed by western blotting. GAPDH was used as a loading control (*n* = 3). Data were shown as mean ± SEM. **p < 0.01 vs. control, ^#^ *p* < 0.05 vs. TGF-β1, ^##^ *p* < 0.01 vs. TGF-β1.

**FIGURE 6:**
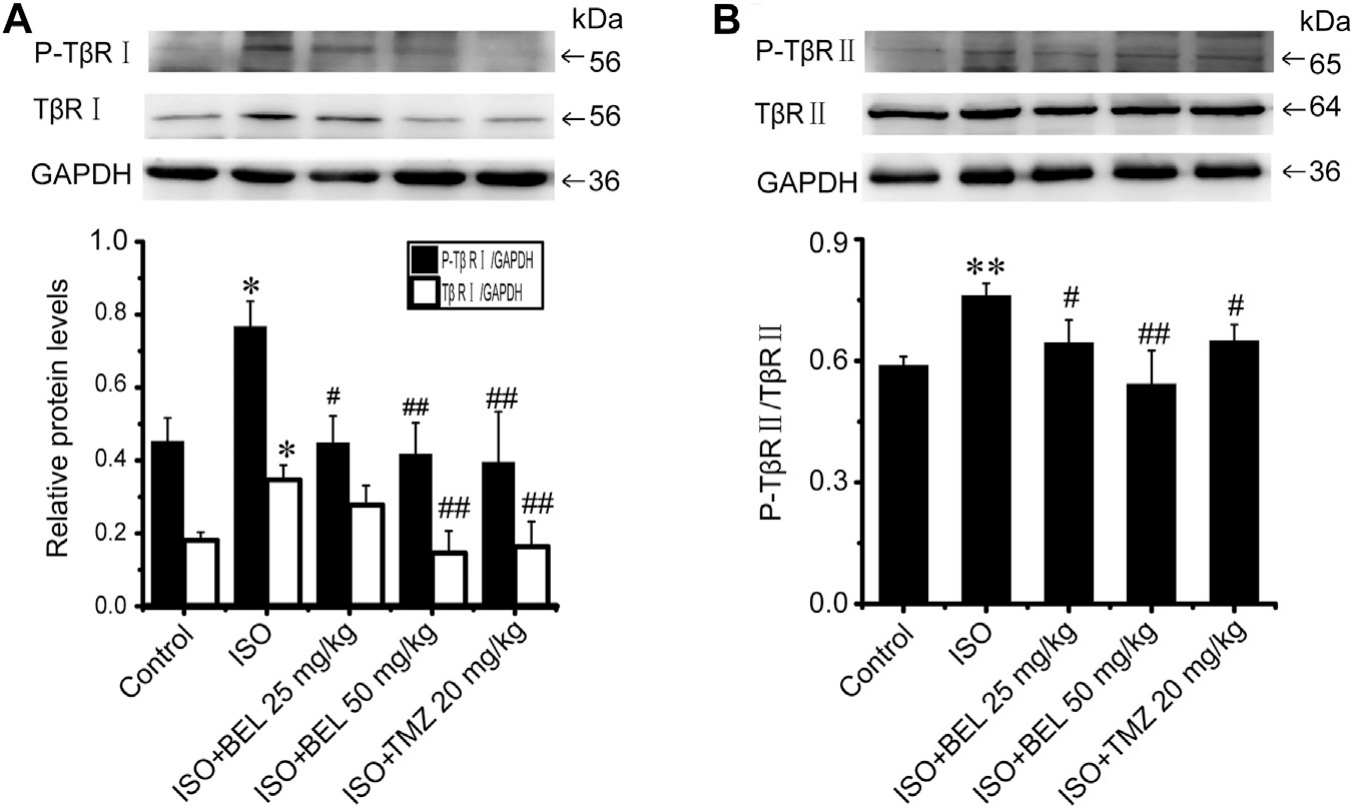
BEL limits the expression of TβRI and the phosphorylation of TβRI and II induced by ISO **(A and B)** Western blotting analysis of TβRII, P-TβRII, TβRI, and P-TβRI. Bar graphs show fold changes of P-TβRI/GAPDH, TβRI/GAPDH and P-TβRII/TβRII (*n* = 3). Data were shown as mean ± SEM. ^*^ *p* < 0.05 vs. control, **p < 0.01 vs. control, ^#^ *p* < 0.05 vs. ISO, ^##^ *p* < 0.01 vs. ISO.

The phosphorylation and activation of TGF-β receptors are key events in the signal transmission of TGF-β. The expression and phosphorylation of TβRⅡ and TβRⅠ were checked by western blotting. In CFs, TGF-β1 induced the phosphorylation and activation of TβRⅡ and TβRⅠ but did not affect the total expression of TβRⅡ and TβRⅠ comparing with the control group; this phosphorylation and activation of TβRs was limited by BEL administration (Figure 5). *In vivo*, the levels of phospho-TβRⅡ and TβRⅠ and TβRⅠ expression were significantly increased in the ISO group vs. the control group. However, ISO-induced increases in TβRⅠ expression and the phospho-TβRⅡ and TβRⅠ levels were decreased by BEL and TMZ (Figure 6). The results indicated that BEL inhibited the phosphorylation and activation of the receptors of TGF-β.

### BEL Regulates Smad Proteins Expression and Phosphorylation

**FIGURE 7:**
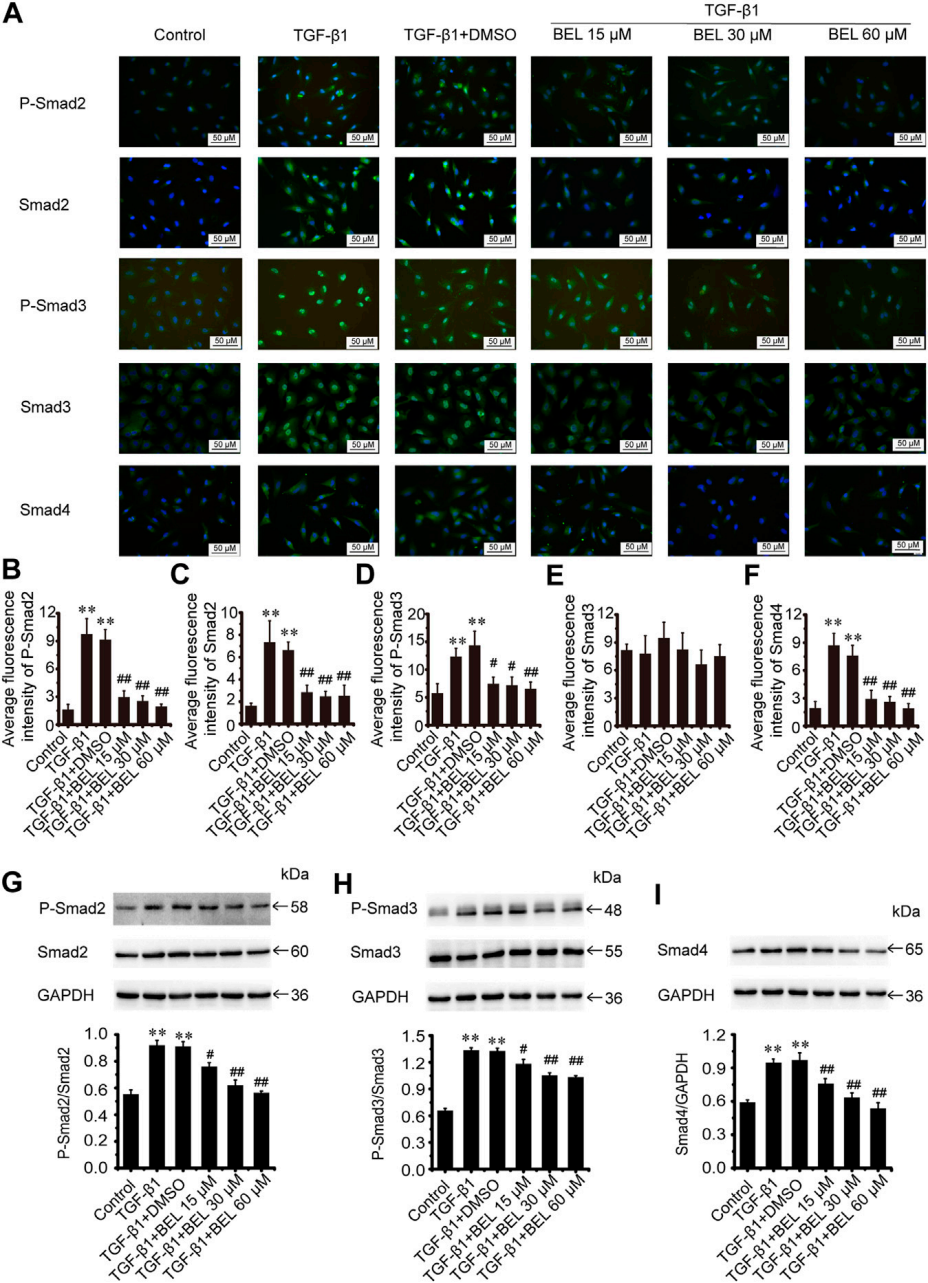
BEL inhibits Smads signaling in CFs **(A)** The effects of different doses of BEL (15, 30, 60 μM) on the expression of Smads2, 3, and 4 and the level of P-Smads2, 3 induced by TGF-β1 using immunofluorescence staining. Cell nuclei was counterstained in blue with DAPI **(B–F)** Bar graphs show quantitative analyses of fluorescent signals for P-Smads2, Smads2, P-Smads2, Smad3, and Smad4 **(G–I)** Western blotting detects Smad proteins expression and phosphorylation levels. Bar graphs show fold changes of P-Smad2/Smad2, P-Smad3/Smad3, and Smad4/GAPDH (*n* = 3). Data were shown as mean ± SEM. **p < 0.01 vs. control, ^#^ *p* < 0.05 vs. TGF-β1, ^##^ *p* < 0.01 vs. TGF-β1.

**FIGURE 8:**
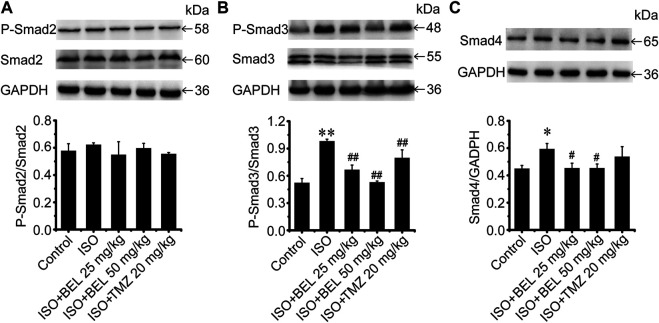
BEL regulates Smad proteins expression and phosphorylation *in vivo* **(A–C)** Western blotting analysis of Smads2, 3, 4 expression and Smads2, 3 phosphorylation levels. Bar graphs show fold changes of P-Smad2/Smad2, P-Smad3/Smad3 and Smad4/GAPDH (*n* = 3). Data were shown as mean ± SEM. *p < 0.05 vs. control, **p < 0.01 vs. control, ^#^ *p* < 0.05 vs. ISO, ^##^ *p* < 0.01 vs. ISO.

The TGF-β1/Smads signaling pathway plays a critical role in myocardial fibrosis (Hu et al., 2018). The effect of BEL on Smad proteins was investigated in the context of myocardial fibrosis. *In vitro*, immunofluorescence staining showed that the expression of Smads2/4 and the levels of phospho-Smads2, 3 were significantly elevated by TGF-β1 stimulation vs. the control group. These elevations were inhibited by BEL treatment (Figures 7A–F). Consistent with immunofluorescence staining, western blotting analysis indicated that BEL inhibited the expression of Smads2, 4 and the levels of Smads2, 3 phosphorylation (Figures 7G–I). *In vivo*, the results showed that the expression of Smad4 and the level of phospho-Smad3 were significantly elevated in the ISO group vs. the control group. However, BEL treatment decreased the Smad4 expression and the Smad3 phosphorylation level compared with the ISO group (Figure 8). These results demonstrated that BEL treatment inhibited the expression of Smads2 and 4 and the activation of Smads2 and 3 in CFs. These results indicated that BEL could inhibit TGF-β/Smads signaling pathway to alleviate myocardial fibrosis in CFs.

### BEL Prevents TGF-β1-Induced p38 Phosphorylation in CFs

**FIGURE 9:**
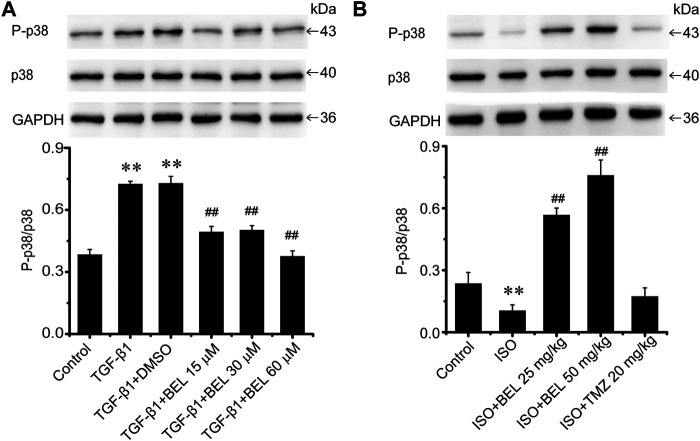
The effects of BEL on p38 signaling *in vitro* and *in vivo* **(A)** The effects of different doses of BEL (15, 30, 60 μM) on the expression p38 and the level of P-p38 in CFs induced by TGF-β1 using western blotting. Bar graphs show fold changes of P-p38/p38 (*n* = 3). Data were shown as mean ± SEM. **p < 0.01 vs. control, ^##^ *p* < 0.01 vs. TGF-β1 **(B)** The effects of BEL on the expression p38 and the level of P-p38 *in vivo* by western blotting. Bar graphs show fold changes of P-p38/p38 (*n* = 3). Data were shown as mean ± SEM. **p < 0.01 vs. control, ^##^ *p* < 0.01 vs. ISO.

Besides TGF-β/Smads canonical signaling, p38 plays an important role in fibrosis (Hu et al., 2016; Liu et al., 2017). The levels of p38 expression and phosphorylation were examined by western blotting in the cultured CFs. The results showed that p38 was significantly phosphorylated and activated by TGF-β1 stimulation. However, BEL treatment inhibited the activation of p38, but the expression of total p38 did not change (Figure 9A). Interestingly, in ISO-induced injury heart tissue, the level of p38 phosphorylation was downregulated vs. the control group; the downregulation was upregulated by BEL treatment but not TMZ administration (Figure 9B). These results illustrated that BEL prevented the phosphorylation of p38 in CFs.

### BEL Inhibits Smad3 Phosphorylation Independent of p38 in CFs

**FIGURE 10:**
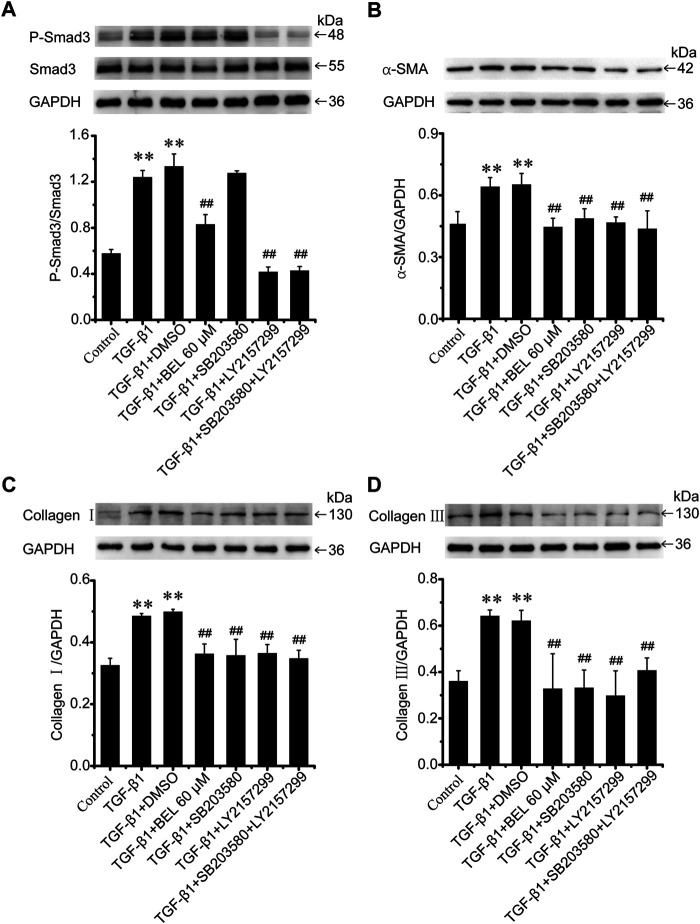
BEL inhibits Smad3 phosphorylation independent of p38 in CFs **(A)** The expression of Smad3 and the level of P-Smad3 by western blotting. Bar graphs show fold changes of P-mad3/Smad3 (*n* = 3) **(B–D)** Expression of α-SMA, collagen Ⅰ, and collagen Ⅲ in CFs. Bar graphs show fold changes for the collagen Ⅰ, collagen Ⅲ, and α-SMA expression as analyzed by western blotting. GAPDH was used as a loading control (*n* = 3). Data were shown as mean ± SEM. **p < 0.01 vs. control, ^##^ *p* < 0.01 vs. TGF-β1.

BEL may downregulate the levels of phospho-p38 and phospho-Smad3; but it is unclear whether the inhibitory roles of BEL on p38 signaling suppress Smad3 activation. CFs were pretreated with DMSO, BEL (60 µM), SB203580 (0.5 µM), and LY2157299 (10 µM) or SB203580 plus LY2157299 for 0.5 h. They were then stimulated with TGF-β1 for 24 h. Western blotting analyzed Smad3 expression and phospho-Smad3 level. The results showed that TGF-β1-induced an increase in Smad3 phosphorylation that was significantly downregulated by BEL, LY2157299, and SB203580 plus LY2157299 but not SB203580 (Figure 10A). Further, the expression of α-SMA, collagen Ⅰ, and collagen Ⅲ were detected. The results showed that the elevations in α-SMA, collagen Ⅰ, and collagen Ⅲ induced by TGF-β1 were decreased by BEL, SB203580, LY2157299, and SB203580 plus LY2157299 (Figures 10B–D). The inhibition of p38 does not affect phosphorylation of Samd3. This study indicated that BEL could inhibit phenotypic transformation of CFs by inhibiting the canonical TGF-β pathway and non-canonical signaling pathway respectively.

### BEL Impedes NR4A1 From the Nucleus to Cytoplasmic Localization

**FIGURE 11:**
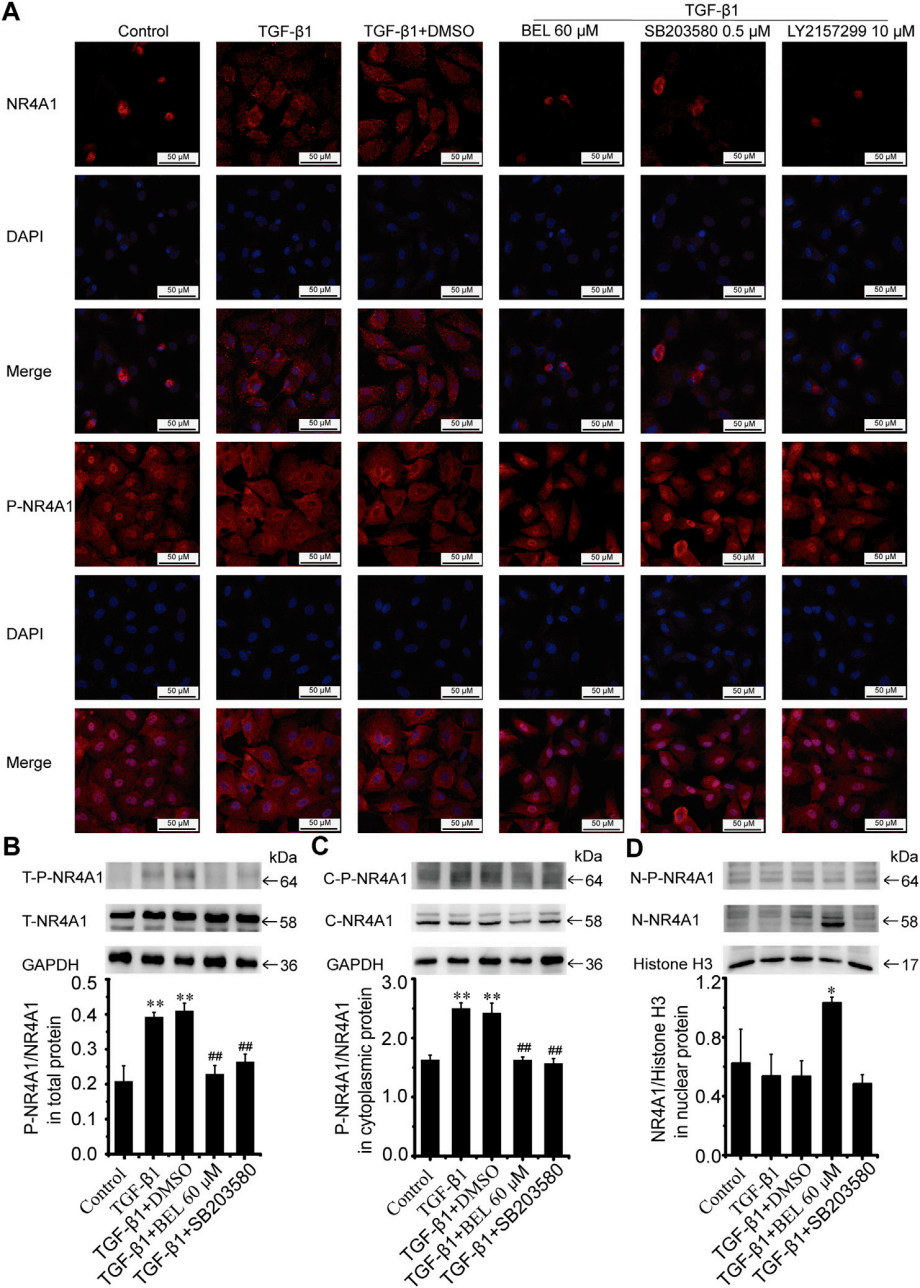
BEL impedes NR4A1 from the nucleus to cytoplasmic localization **(A)** Effects of BEL, SB203580 and LY2157299 on the expression of NR4A1 and P-NR4A1 induced by TGF-β1 using immunofluorescence staining. Cell nuclei was counterstained in blue with DAPI **(B)** Expression of NR4A1 and the levels of P-NR4A1 in total protein (T-NR4A1, T-P-NR4A1) by western blotting. Bar graphs show fold changes of P-NR4A1/NR4A1 (*n* = 3) **(C)** Expression of NR4A1 and the levels of P-NR4A1 in cytoplasmic protein (C-NR4A1, C-P-NR4A1) by western blotting. Bar graphs show fold changes of P-NR4A1/NR4A1 (*n* = 3) **(D)** Expression of NR4A1 and the levels of P-NR4A1 in nuclear protein (N-NR4A1, N-P-NR4A1) by western blotting. Bar graphs show fold changes of NR4A1/H3 (*n* = 3). Data were shown as mean ± SEM. **p < 0.01 vs. control, ^#^ *p* < 0.05 vs. TGF-β1, ^##^ *p* < 0.01 vs. TGF-β1.

NR4A1 is a feedback molecule for regulating TGF-β signaling. In fibrotic disease, NR4A1 transferred from the nucleus to the cytoplasm losing its negative feedback regulation effect on TGF-β signaling. At the same time, p38 activation promotes the transformation of NR4A1 from the nucleus to the cytoplasm. To illuminate the relationship of NR4A1 and p38 signaling, CFs were pretreated with BEL or SB203580 for 0.5 h then stimulated with TGF-β1 for 24 h. Western blotting analysis demonstrated that BEL and SB203580 inhibited TGF-β1-induced the upregulation of phospho-NR4A1 (Figure 11B). Immunofluorescence staining indicated that TGF-β1 promoted NR4A1 and phospho-NR4A1 cytoplasmic localization while pretreatments with BEL and SB203580 limited phospho-NR4A1 transformation (Figure 11A). Further western blotting showed NR4A1 expression and phosphorylation levels in the cytoplasm and nucleus. The results illustrated that phospho-NR4A1 level was enhanced in the cytoplasm by TGF-β1. However, BEL and SB203580 decreased the enhancements of phospho-NR4A1 in the cytoplasm (Figure 11C), and BEL also enhanced nuclear retention of NR4A1 (Figure 11D). These data illustrated that BEL impeded NR4A1 cytoplasmic localization and restored the activation of NR4A1 to inhibit TGF-β1 signaling. Therefore, the inhibitory role of BEL on CFs activation was exerted by suppressing NR4A1 from the nucleus to cytoplasmic localization.

In summary, our data suggest that BEL can ameliorate myocardial fibrosis by inhibiting the proliferation and phenotypic transformation of CFs; and these inhibitory effects of BEL are related to the regulation of TGF-β/Smads and p38 pathway and prevention of NR4A1 cytoplasmic localization that negatively regulated TGF-β signaling in CFs.

## Discussion

*G. acuta*, a kind of natural plant of Gentianaceae, has been used as a health tea to clear the heat and toxic materials, remove pathogenic heat from blood, and promote the secretion of urine in the Hulunbeier districts of Inner Mongolia. Ewenki people keep a habit of drinking “guixincao” tea, and employ it to treat angina by water decoction or chewing for a long time (Wunir et al., 2009; Li et al., 2010). Our previous studies demonstrated that *G. acuta* could alleviate ISO-induced acute and chronic myocardial injury, and ameliorate myocardial fibrosis (Li et al., 2019; Sun et al., 2021; Yang et al., 2020).

An increasing body of evidence suggests that the xanthones isolated from *G. acuta* protect the heart from injury (Wang et al., 2017; Wang et al., 2018). BEL is the main component of xanthones in *G. acuta.* Research has shown that BEL could alleviate myocardial ischemia-reperfusion injury and inhibit H_2_O_2_-induced apoptosis on H9c2 cells, but the inhibitory role of BEL on myocardial fibrosis is not clear. Here, the potential role of BEL in treatment of myocardial fibrosis was investigated *in vivo* and *in vitro* using a mouse myocardial fibrosis model and TGF-β1-induced CFs phenotypic transformation model.

CFs are the main effector cells of the heart in response to injury. They are involved in the development of cardiac remodeling following MI. Myocardial fibrosis was induced after MI by promoting CFs proliferation and activation, and inhibition of CFs proliferation and activation could improve myocardial fibrosis (Virag and Murry, 2003; Travers et al., 2016; Wang et al., 2019). *In vitro* studies have shown that TGF-β1 can promote the proliferation and phenotypic transformation of CFs by activating TGF-β signaling pathway, and inhibition of TGF-β1-induced myofibroblast differentiation can be an important therapeutic strategy for myocardial fibrosis (Wang et al., 2018; Zhang et al., 2018; Wang et al., 2019; Zhou et al., 2019).

CFs are activated and transform into myofibroblasts during pathological tissue repair. Myofibroblasts can express a contractile protein such as α-SMA and produce excessive collagen during fibrosis progression (Weber and Diez, 2016; Song et al., 2020). Our previous studies demonstrated that TGF-β1 expression was upregulated in fibrotic heart tissue, and *G. acuta* downregulated the upregulation and ameliorated myocardial fibrosis by inhibiting TGF-β1 signaling (Li et al., 2019; Yang et al., 2020). This work evaluated the effect of BEL, a vital component of *G. acuta*, on cell viability with or without TGF-β1. The results showed that BEL inhibited TGF-β1-induced CFs proliferation but did not affect cells viability without TGF-β1. The α-SMA expression was increased by TGF-β1 stimulation, and BEL treatment decreased the increase of α-SMA induced by TGF-β1 in CFs. BEL also inhibited the excessive expression of collagen Ⅰ and collagen Ⅲ. These results indicated that BEL inhibited TGF-β1-induced myofibroblast differentiation. *In vivo* studies indicated that BEL treatment ameliorated the cardiac disorder structure and myocardial fibrosis by inhibiting ISO-induced increases in α-SMA, collagen Ⅰ, and collagen Ⅲ while preventing collagen deposition in fibrotic tissues. These results demonstrated that these inhibitory roles of BEL on myocardial fibrosis were exerted by inhibiting the proliferation and phenotypic transformation of CFs.

The molecular mechanisms underlying myocardial fibrosis are complex and include a series of intracellular signal transduction processes. TGF-β1 is an important profibrotic cytokine involved in fibrosis. It activates its downstream specific serine/threonine kinase receptors to regulate tissue fibrosis and tissue scarring (Xie et al., 2017). TGF-β1 receptors phosphorylation and activation play a primary role in the intracellular signal transduction process (Dobaczewski et al., 2011). This study showed that BEL treatment inhibited the levels of phosphorylated TβRⅡ and TβRⅠ elevated by TGF-β1 stimulation in CFs. BEL also decreased TβRⅠ expression and the levels of phosphorylated TβRⅡ and TβRⅠ induced by ISO *in vivo*. These results demonstrated that BEL could prevent the phosphorylation and activation of TGF-β receptors thereby inhibiting intracellular signal transduction process from ameliorating myocardial fibrosis.

Studies have shown that Smads canonical signaling pathways contributed to TGF-β-induced cardiac fibrosis (Li et al., 2019; Zhou et al., 2019). One study reported that TGF-β-Smad2/3 signaling in activated tissue-resident cardiac fibroblasts is a principal mediator of the fibrotic response, and the deletion of Smad2/3 in activated cardiac fibroblasts blocked the expression of fibrosis marker genes such as α-SMA, collagen Ⅰ, and collagen Ⅲ (Khalil et al., 2017). In CFs, BEL administration reduced TGF-β1-induced expression of Smads2 and 4 and significantly inhibited the levels of Smads2 and 3 phosphorylation. In fibrotic heart tissue, BEL treatment suppressed Smad3 phosphorylation and decreased Smad4 expression but did not inhibit Smad2 expression and phosphorylation. The data suggest that BEL could mediate TGF-β/Smads canonical signaling in CFs and especially impede TGF-β/Smad3 activation, which is the most major profibrotic signaling element. This can suppress the expression of α-SMA, collagen Ⅰ, and collagen Ⅲ.

The p38 MAPK is a vital family member of the MAPK cascade and is a non-canonical pathway regulated by TβRⅡ. Current research has shown that p38 is a dominant regulator in myocardial fibrosis. Some studies have shown that TGF-β may induce activation of p38, and the prevention of p38 could alleviate fibrosis (Yamashita et al., 2008; Kojonazarov et al., 2017). Activin A promoted CF proliferation and collagen production by activating p38 while inhibition of p38 prevented activin A-regulated responses (Hu et al., 2016). This study demonstrated that BEL decreases the phosphorylation of p38 induced by TGF-β1 in CFs. Interestingly, the level of p38 phosphorylation was downregulated in the heart tissue injury induced by ISO whereas the downregulation was upregulated by BEL treatment.

In the heart, there are many cell types including cardiomyocyte and CFs. A report has shown that noradrenaline activated p38 MAPK through its interaction with α-adrenoceptors in cardiomyocytes, which could protect against cardiomyocyte apoptotic cell death. In contrast, ISO decreased the phosphorylation of p38 MAPK through its interaction with β-adrenoceptors in cardiomyocytes (Tsang and Rabkin, 2009). However, in CFs, TGF-β1 can stimulate p38 signaling activation (Meyer-Ter-Vehn et al., 2006). This distinct change in p38 *in vivo* and *in vitro* may be due to different responses of cardiomyocytes and CFs to ISO. This *in vivo* result was consistent with another report in which increasing p38 activation could improve functional recovery (Fan et al., 2010).

This study showed that BEL inhibited myocardial fibrosis by regulating TGF-β/Smads and p38 signaling. Activation of p38 could increase the Smad3 phosphorylation in hepatic fibrosis (Fabre et al., 2018). Here, the relation of p38 signaling and Smad3 phosphorylation was further investigated in CFs. The results demonstrated that inhibition of p38 did not affect the phosphorylation of Smad3 but could significantly reduce TGF-β1-induced expression of α-SMA, collagen Ⅰ, and collagen Ⅲ. The inhibitory effect of BEL on CFs activation was via inhibition of the canonical TGF-β pathway and non-canonical signaling pathway respectively.

NR4A1 is an endogenous inhibitor of TGF-β signaling, which can limit profibrotic TGF-β1 effects thereby identifying it as a potential target for anti-fibrotic therapies. In fibrotic diseases, NR4A1 is phosphorylated and located in the cytoplasm and lost the inhibitory role on TGF-β1 signaling (Palumbo-Zerr et al., 2015). Prevention of NR4A1 transferring from nucleus to cytoplasm might alleviate fibrosis (Zeng et al., 2018). NR4A1 interaction with p38 promoted p65 activation and elevated the levels of inflammatory cytokines in LPS-induced inflammation (Li et al., 2015). It has been found that NR4A1 can be transferred to cytoplasm, mitochondria and/or endoplasmic reticulum (ER) under certain stimulation (Pawlak et al., 2015). In the study of ALA-induced apoptosis of VSMCs, prevention of p38 signaling impeded NR4A1 cytoplasmic localization (Kim et al., 2010). Here, immunofluorescence staining and western blotting analysis illustrated that TGF-β1 obviously induced NR4A1 phosphorylation and increased NR4A1 cytoplasmic localization. However, these increases in phospho-NR4A1 were suppressed by BEL and a p38 inhibitor. And BEL could enhance nuclear retention of NR4A1. A recent study demonstrated that TGF-β-induced nuclear export of NR4A1 is dependent on activation of the p38 MAPK pathway in breast cancer cells (Hedrick and Safe 2017). However, another report showed that TGF-β-induced nuclear export of NR4A1 in lung cancer cells is JNK-dependent and not p38 dependent (Hedrick et al., 2018). From the above, the NR4A1 nuclear output is associated with phosphorylation and varies with cell type. Our results suggested that nuclear retention of NR4A1 was enhanced by BEL not SB203580, that may be related to inhibition of NR4A1 phosphorylation. Our results verified that BEL prevented myocardial fibrosis by inhibiting NR4A1 phosphorylation and impeding NR4A1 nucleus to cytoplasmic transformation in CFs.

In this study, the inhibitory effect of BEL on myocardial fibrosis has been confirmed for the first time, and the relevant molecular mechanism has been clarified, which may be related to the inhibition of the proliferation and phenotypic transformation of CFs by regulating TGF-β1/Smads and p38 signaling and preventing NR4A1 cytoplasmic localization. Additionally, there are the following problems, such as the differences between p38 phosphorylation *in vitro* and *in vivo*, the relationship between p38 activation and nuclear NR4A1 output, the detailed mechanism of BEL’s inhibitory effect on NR4A1 nuclear output in CFs and its effect on p38 signaling in other cells, which need to be confirmed by further research.

## Conclusion

**FIGURE 12:**
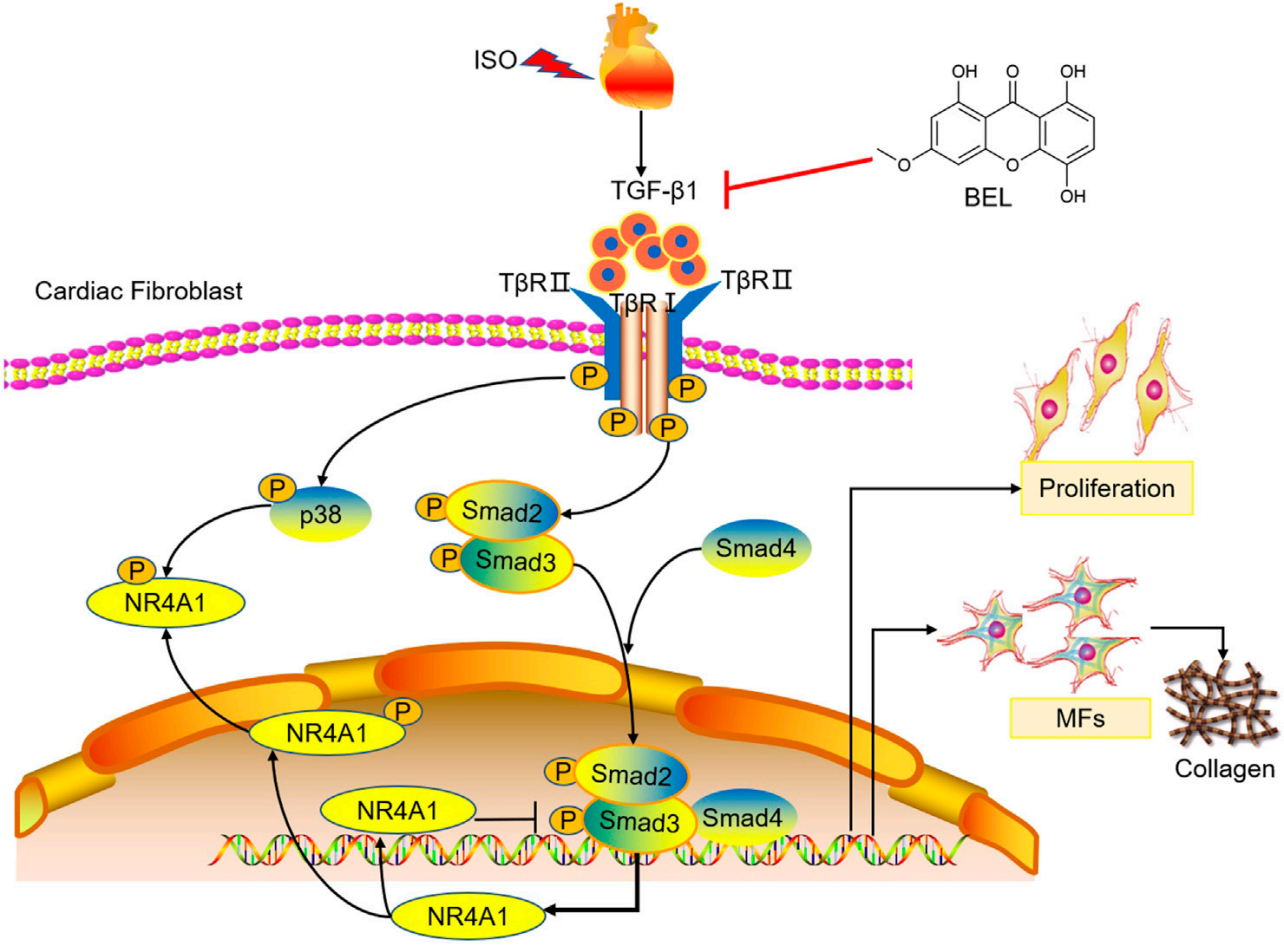
The proposed antifibrotic mechanism of BEL. BEL downregulates the phosphorylation of TβRI and TβRII, suppresses Smads2 and 4 expression and the phosphorylation and activation of Smads2 and 3 in CFs. Furthermore, BEL inhibits p38 phosphorylation and impedes NR4A1 cytoplasmic localization, which restores the inhibitory effect of NR4A1 to TGF-β1-profibrotic signaling. These inhibitory effects further caused a decrease in the expression of α-SMA, collagen I, and collagen III, thereby preventing proliferation and activation of CFs to alleviate myocardial fibrosis.

Overall, both cell and mouse studies verified that BEL protects against myocardial fibrosis by inhibiting the proliferation and phenotypic transformation of CFs. These inhibitory effects might be related to regulating TGF-β1/Smads canonical pathway and p38 MAPK signaling and impeding NR4A1 cytoplasmic localization to restore the inhibitory effect of NR4A1 to TGF-β1 signaling (Figure 12). These results provide new insights into the mechanism underlying the anti-fibrotic effects of BEL in the heart.

## Data Availability

The original contributions presented in the study are included in the article/Supplementary Material, further inquiries can be directed to the corresponding authors.
