# Supplementary material for: Bellidifolin Ameliorates Isoprenaline-Induced Myocardial Fibrosis by Regulating TGF-β1/Smads and p38 Signaling and Preventing NR4A1 Cytoplasmic Localization
Source: Front Pharmacol. 2021 Apr 30;12:644886. doi: 10.3389/fphar.2021.644886 (PMC8120298; doi:10.3389/fphar.2021.644886)
Supplement: Supplementary file 1 [file datasheet1.pdf]

Bellidifolin ameliorates myocardial fibrosis by regulating TGF-β1/Smads and p38 signaling to prevent NR4A1 cytoplasmic localization

Hong-Xia Yang [1,2,†], Jia-Huan Sun [3,†], Ting-Ting Yao [1,†], Yuan Li [1,†], Geng-Rui Xu [1], Chuang Zhang [1], Xing-Chao Liu [4], Wei-Wei Zhou [1], Qiu-Hang Song [1,4,5], Yue Zhang [1,4,5,*], Ai-Ying Li [1,4,5,*]

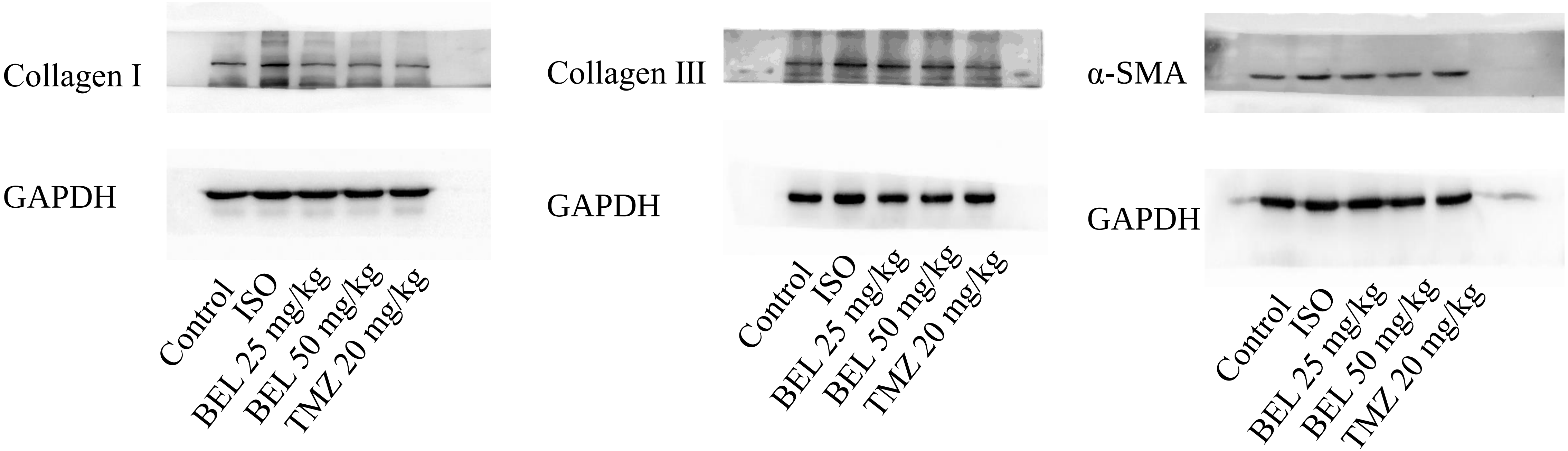
Collagen I
GAPDH
Control
ISO
BEL 25 mg/kg
BEL 50 mg/kg
TMZ 20 mg/kg
Collagen III
GAPDH
Control
ISO
BEL 25 mg/kg
BEL 50 mg/kg
TMZ 20 mg/kg
α-SMA
GAPDH
Control
ISO
BEL 25 mg/kg
BEL 50 mg/kg
TMZ 20 mg/kg

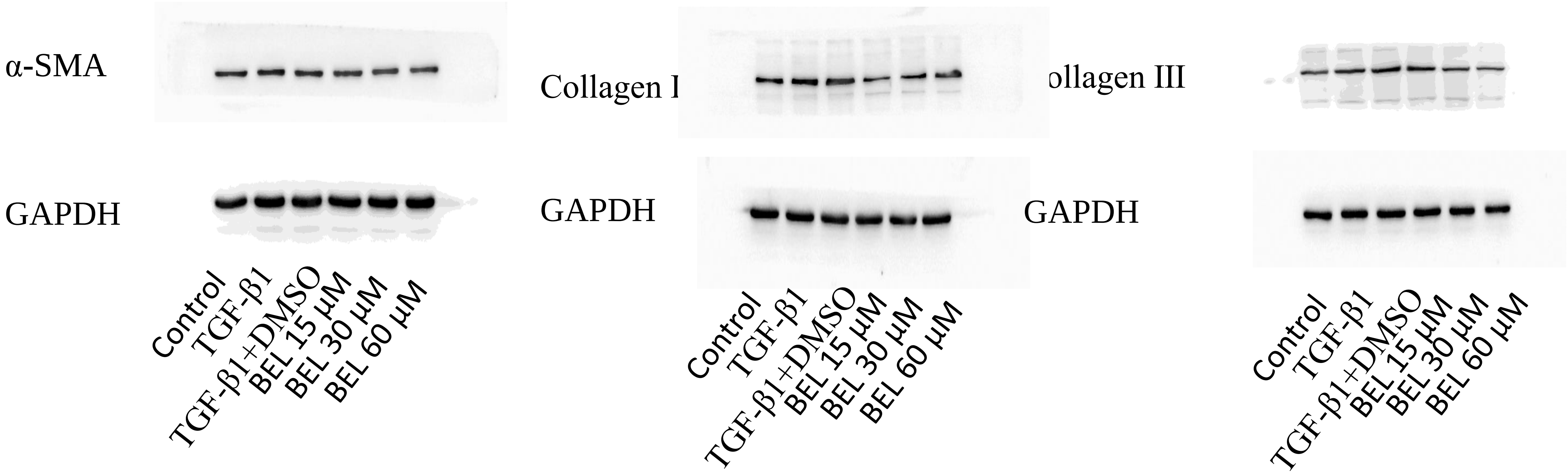
α-SMA
GAPDH
Control
TGF-β1
TGF-β1+DMSO
BEL 15 μM
BEL 30 μM
BEL 60 μM
Collagen I
GAPDH
Control
TGF-β1
TGF-β1+DMSO
BEL 15 μM
BEL 30 μM
BEL 60 μM
ollagen III
GAPDH
Control
TGF-β1
TGF-β1+DMSO
BEL 15 μM
BEL 30 μM
BEL 60 μM

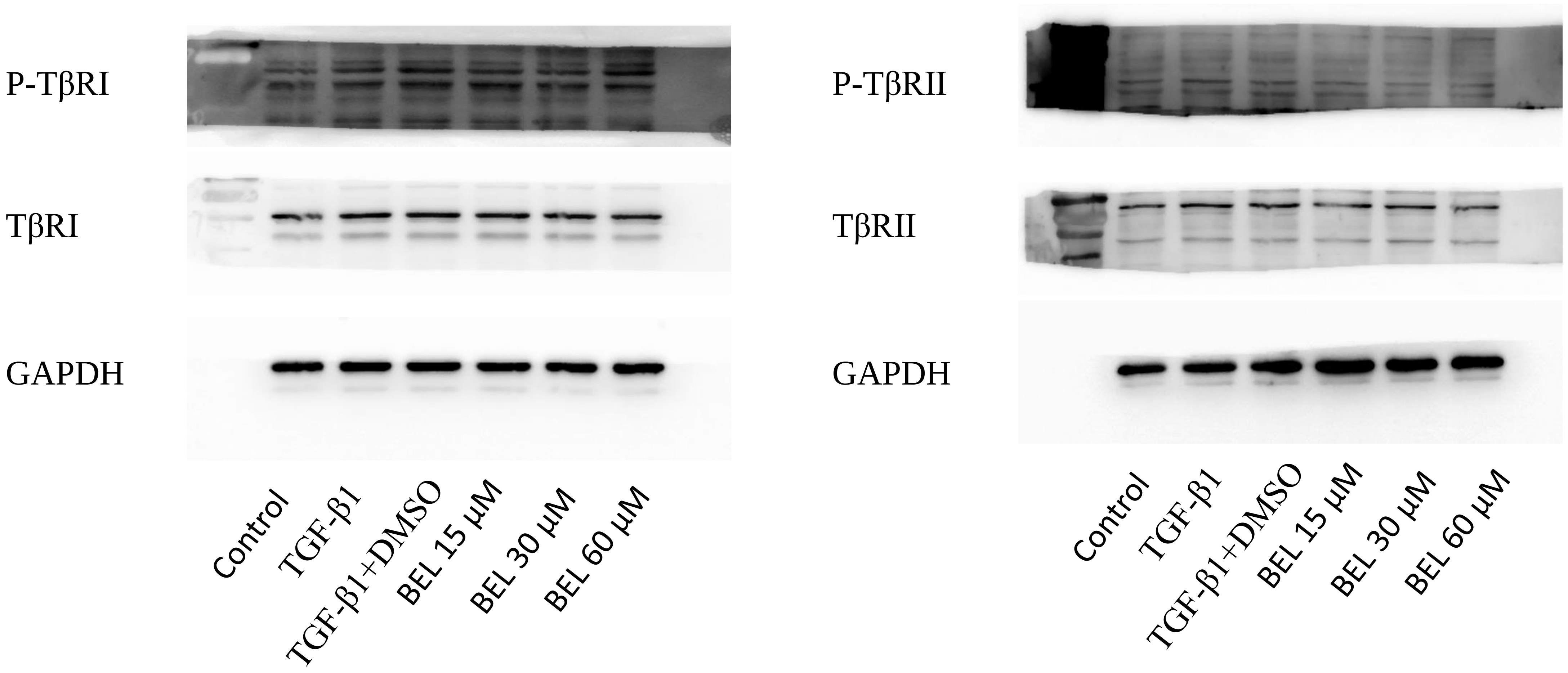

P-TβRI
TβRI
GAPDH
P-TβRII
TβRII
GAPDH
Control
TGF-β1
TGF-β1+DMSO
BEL 15 μM
BEL 30 μM
BEL 60 μM

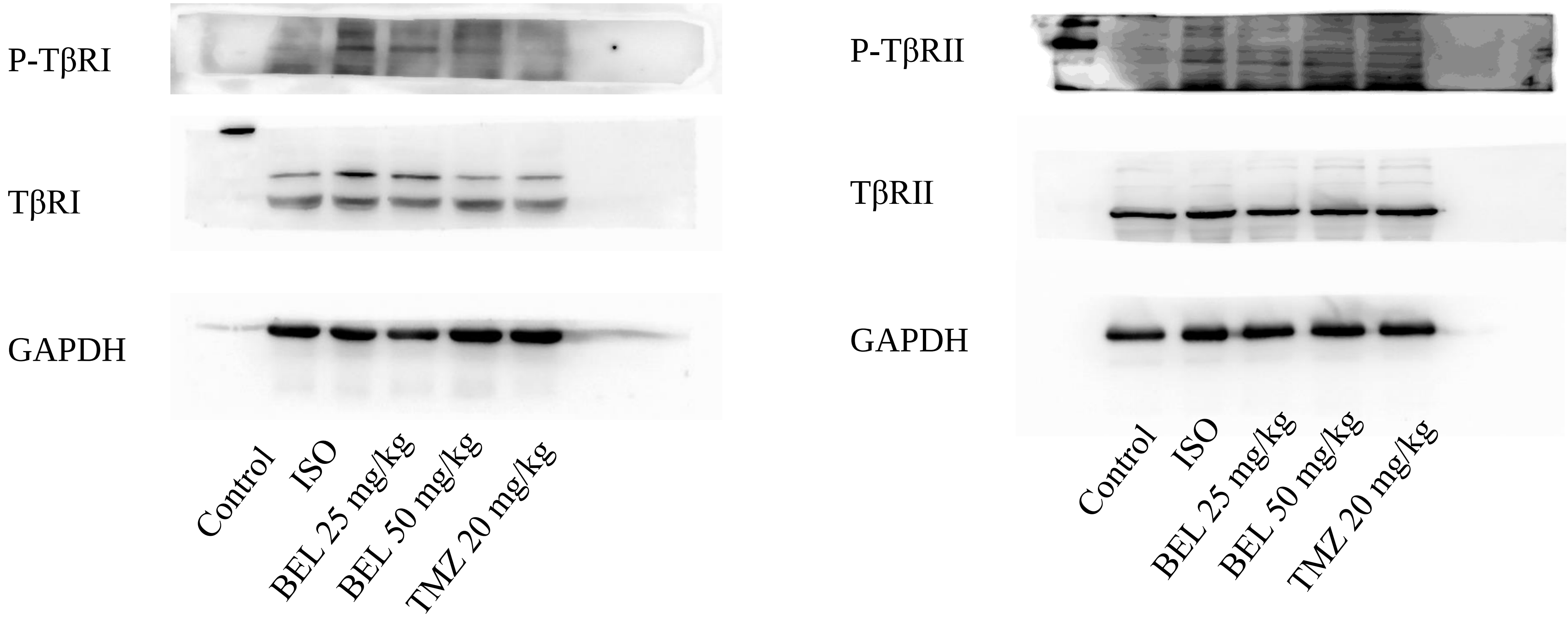

P-TβRI
TβRI
GAPDH
Control
ISO
BEL 25 mg/kg
BEL 50 mg/kg
TMZ 20 mg/kg
P-TβRII
TβRII
GAPDH
Control
ISO
BEL 25 mg/kg
BEL 50 mg/kg
TMZ 20 mg/kg

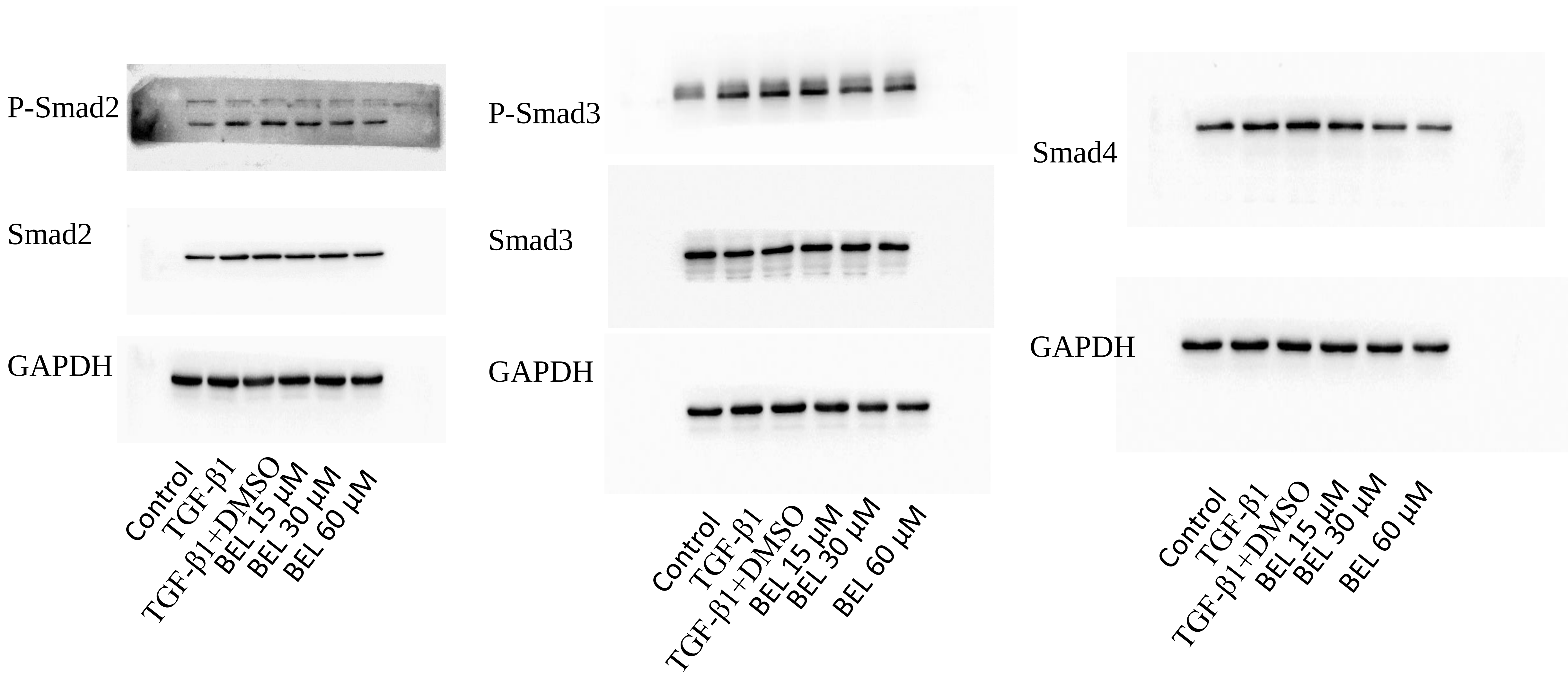
P-Smad2
Smad2
GAPDH
Control
TGF-β1
TGF-β1+DMSO
BEL 15 μM
BEL 30 μM
BEL 60 μM
P-Smad3
Smad3
GAPDH
Control
TGF-β1
TGF-β1+DMSO
BEL 15 μM
BEL 30 μM
BEL 60 μM
Smad4
GAPDH
Control
TGF-β1
TGF-β1+DMSO
BEL 15 μM
BEL 30 μM
BEL 60 μM

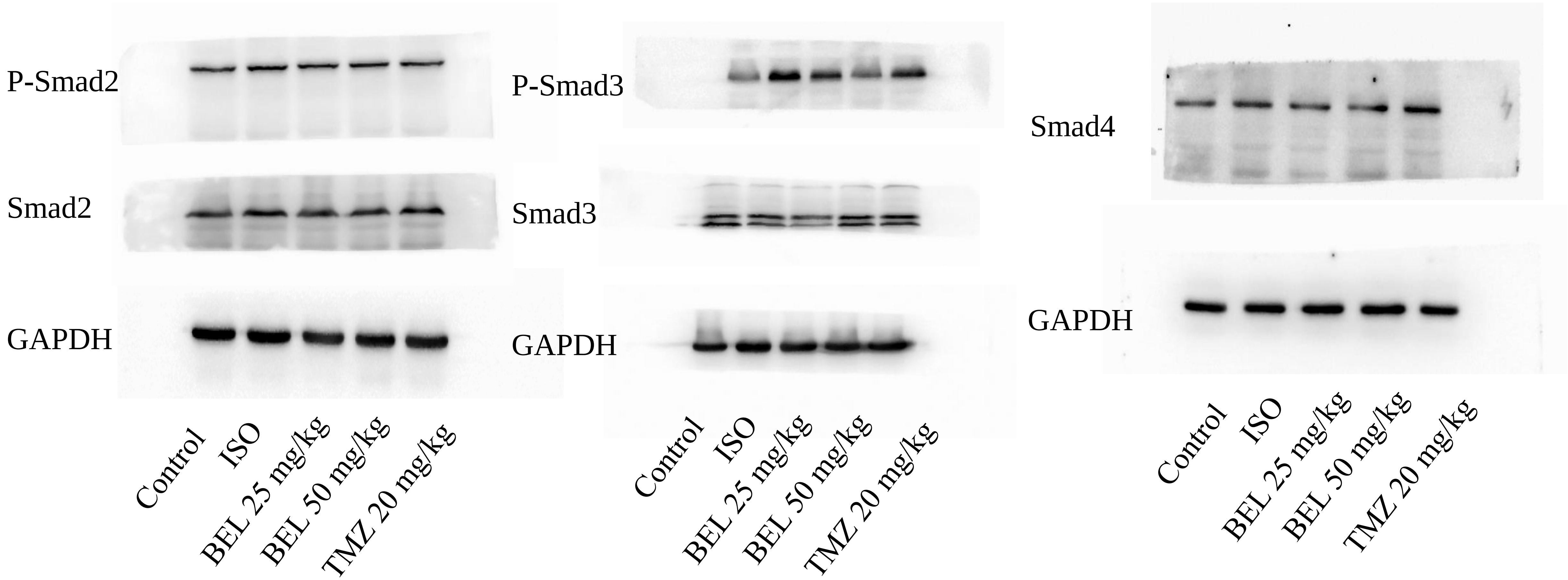
P-Smad2
Smad2
GAPDH
Control
ISO
BEL 25 mg/kg
BEL 50 mg/kg
TMZ 20 mg/kg
P-Smad3
Smad3
GAPDH
Control
ISO
BEL 25 mg/kg
BEL 50 mg/kg
TMZ 20 mg/kg
Smad4
GAPDH
Control
ISO
BEL 25 mg/kg
BEL 50 mg/kg
TMZ 20 mg/kg

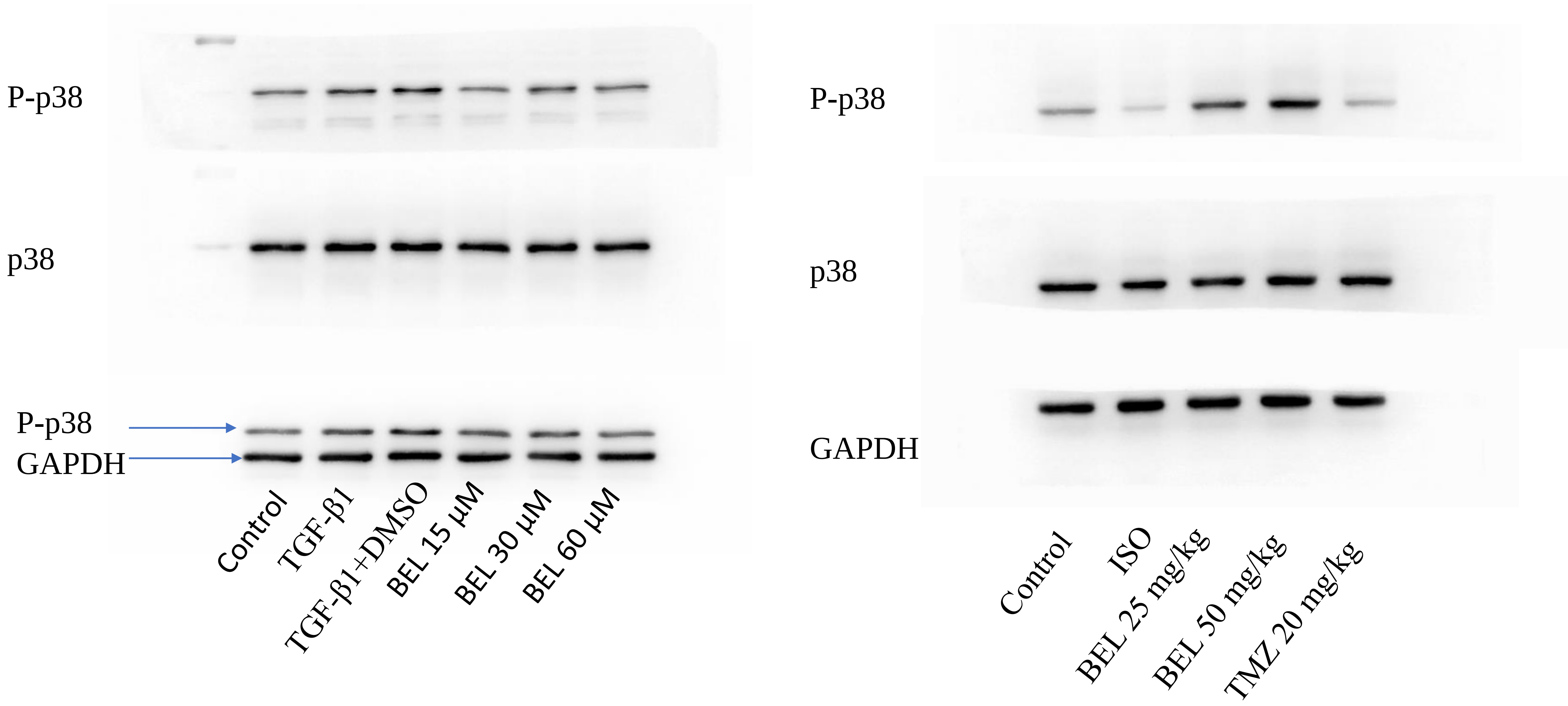

P-p38
p38
P-p38
GAPDH
Control
TGF-β1
TGF-β1+DMSO
BEL 15 μM
BEL 30 μM
BEL 60 μM
P-p38
p38
GAPDH
Control
ISO
BEL 25 mg/kg
BEL 50 mg/kg
TMZ 20 mg/kg

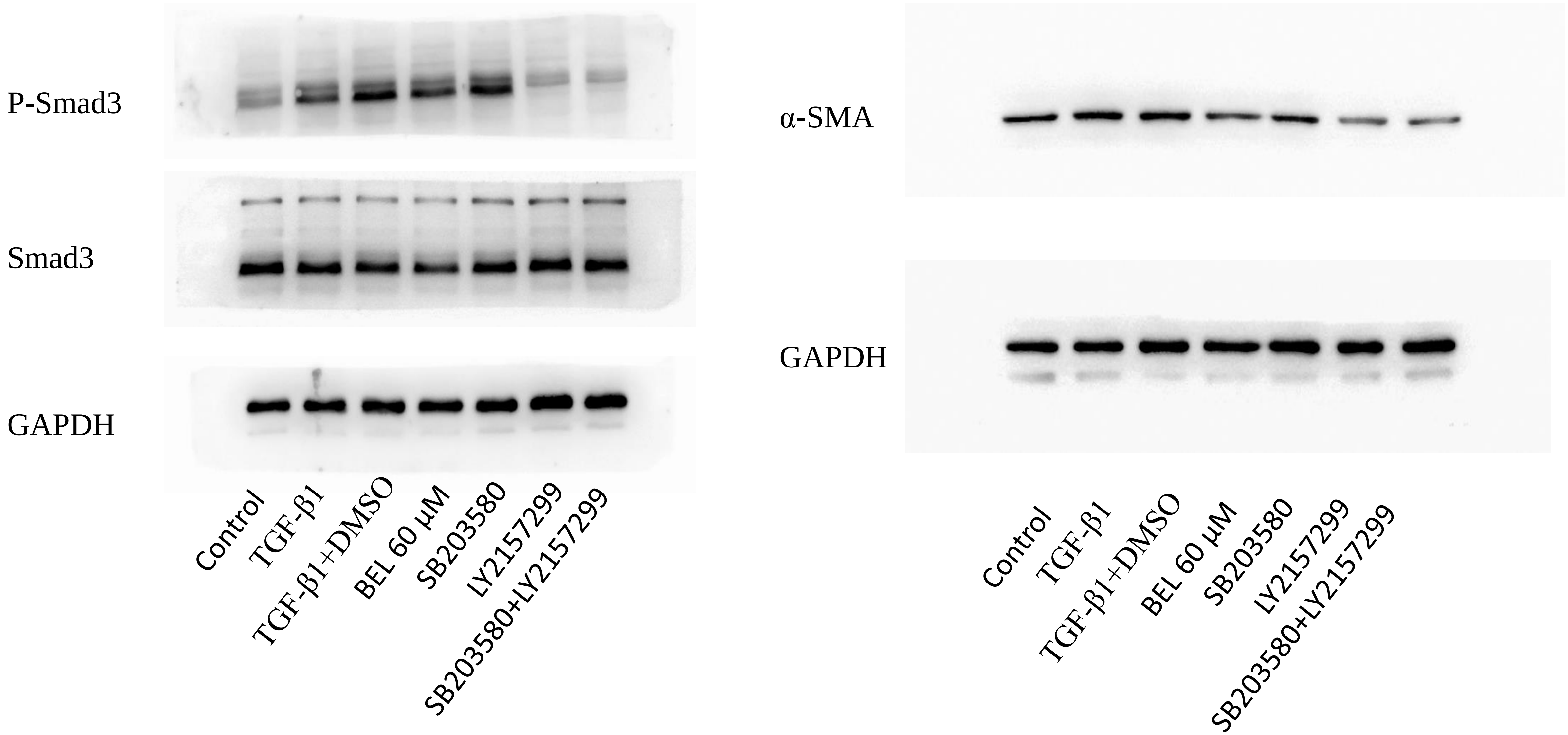

P-Smad3
Smad3
GAPDH
Control
TGF-β1
TGF-β1+DMSO
BEL 60 μM
SB203580
LY2157299
SB203580+LY2157299
α-SMA
GAPDH
Control
TGF-β1
TGF-β1+DMSO
BEL 60 μM
SB203580
LY2157299
SB203580+LY2157299

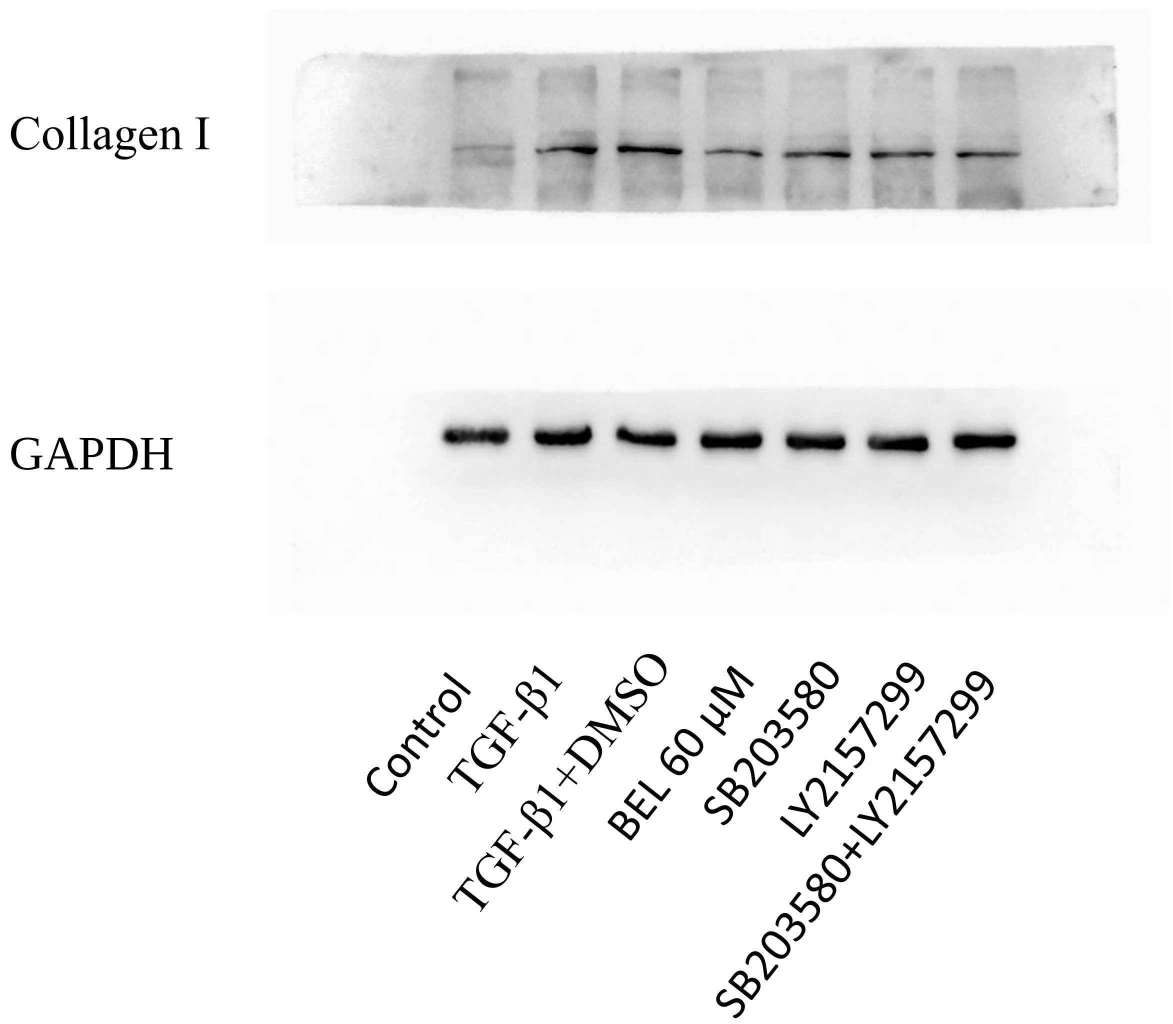

Collagen I
GAPDH
Control
TGF-β1
TGF-β1+DMSO
BEL 60 μM
SB203580
LY2157299
SB203580+LY2157299

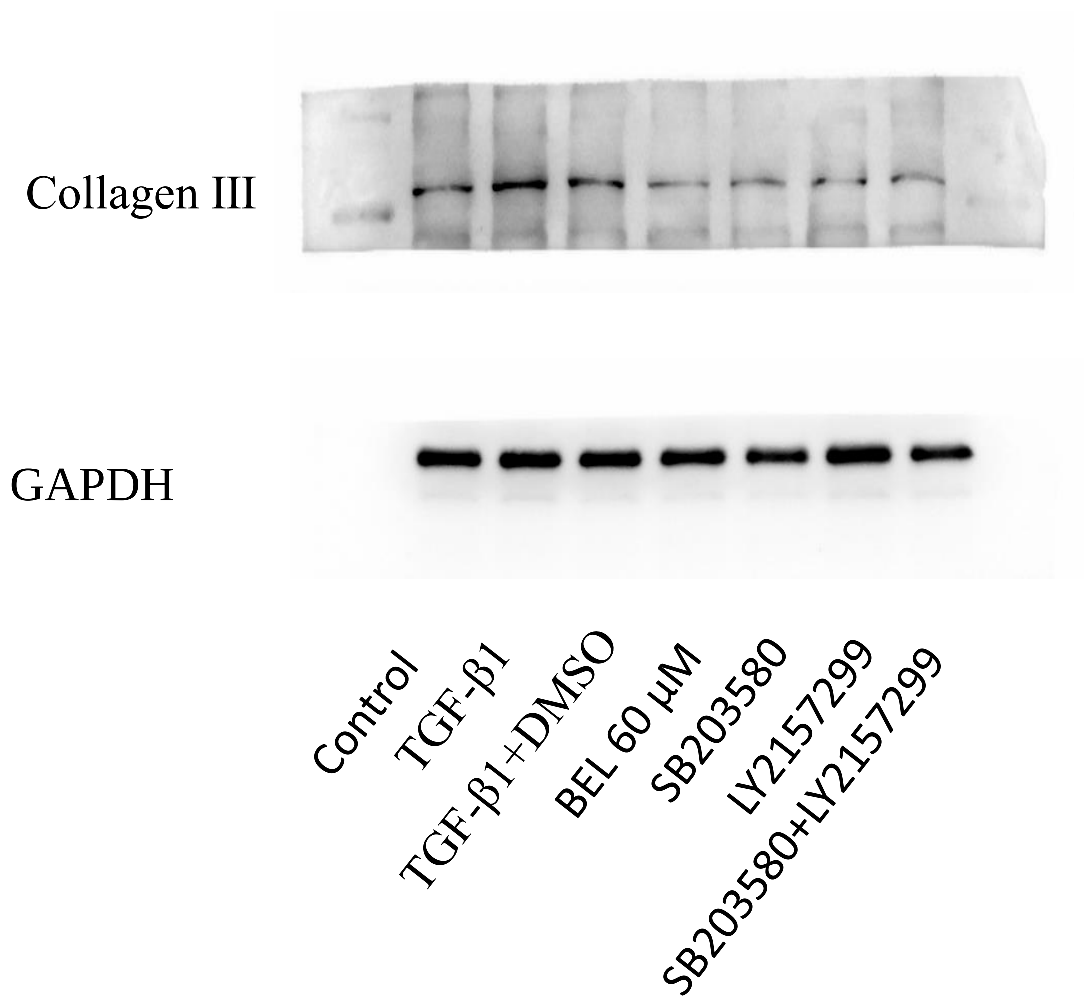

Collagen III
GAPDH
Control
TGF-β1
TGF-β1+DMSO
BEL 60 μM
SB203580
LY2157299
SB203580+LY2157299

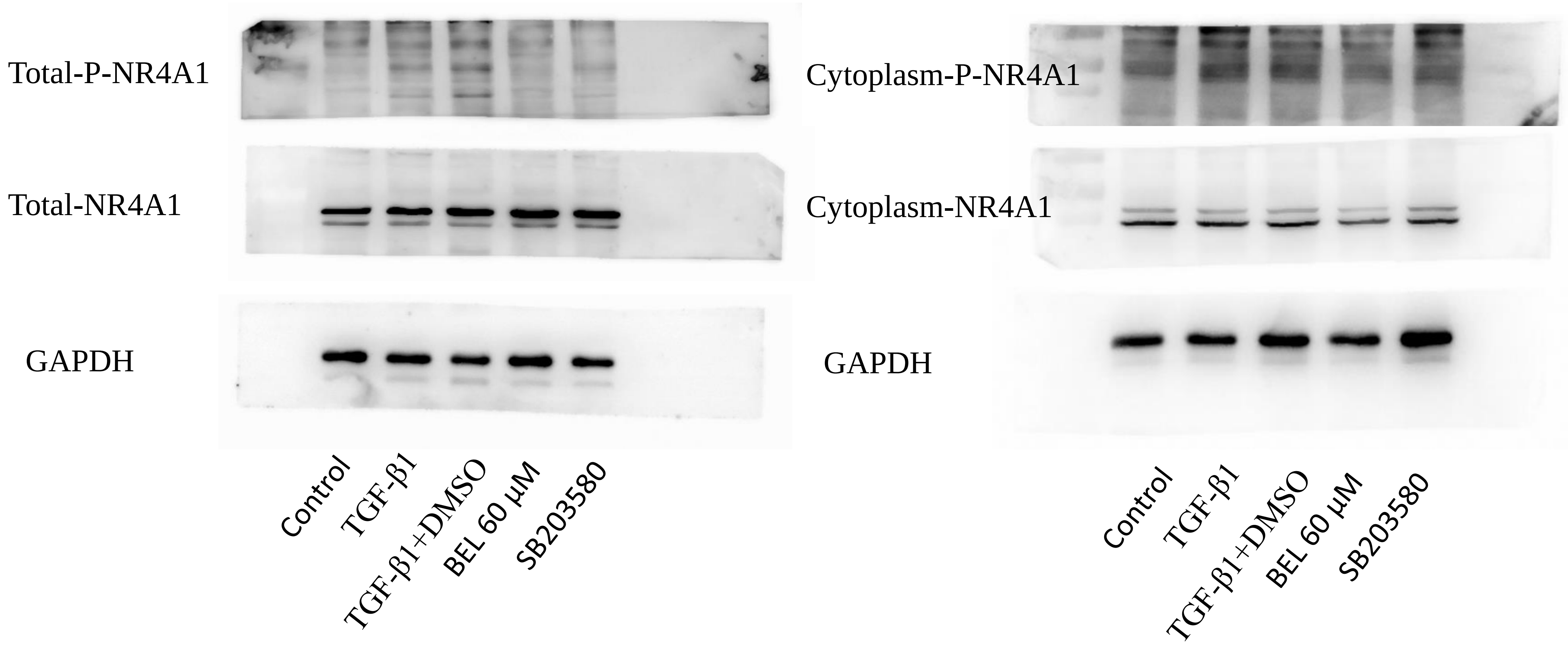
Total-P-NR4A1
Total-NR4A1
GAPDH
Control
TGF-β1
TGF-β1+DMSO
BEL 60 μM
SB203580
Cytoplasm-P-NR4A1
Cytoplasm-NR4A1
GAPDH
Control
TGF-β1
TGF-β1+DMSO
BEL 60 μM
SB203580

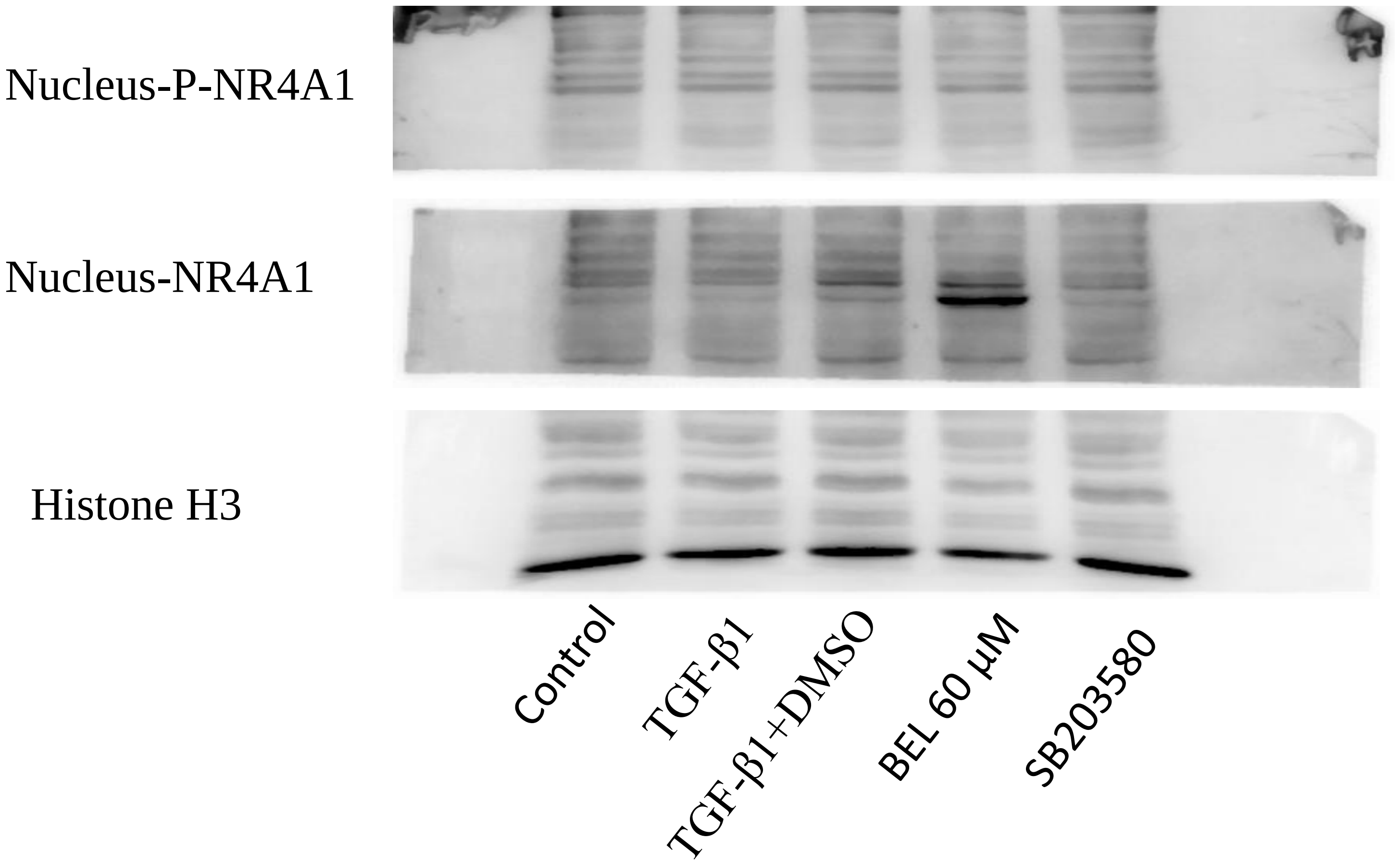

Nucleus-P-NR4A1
Nucleus-NR4A1
Histone H3
Control
TGF-β1
TGF-β1+DMSO
BEL 60 μM
SB203580
